# Epidemiological insights into virulence and antimicrobial resistance of *Escherichia coli* across dairy farms in Nagpur district

**DOI:** 10.3389/fvets.2026.1915714

**Published:** 2026-09-23

**Authors:** Tippa Padmaja, Gayatri Jayaraj, Yashwarya Dharne, Ashwini Tulshiram Nerikar, Shreya Bhojane, S. P. Chaudhari, S. V. Shinde, W. A. Khan, N. V. Kurkure, M. P. Kaore, Rajpal Singh Kashyap, M. S. Patil

**Affiliations:** 1 Department of Veterinary Public Health and Epidemiology, Nagpur Veterinary College, Maharashtra Animal and Fishery Science University, Nagpur, India; 2 Department of Veterinary Pathology, Nagpur Veterinary College, Maharashtra Animal and Fishery Science University, Nagpur, India; 3 Maharashtra Animal and Fishery Science University, Nagpur, India; 4 CIIMS Advanced Research Centre, Nagpur, India; 5 Department of Animal Reproduction, Gynaecology and Obstetrics, Nagpur Veterinary College, Maharashtra Animal and Fishery Science University, Nagpur, India

**Keywords:** *Escherichia coli*, MAR index, multidrug resistance, One Health, organized dairy farms, seasonal variation, virulence

## Abstract

Antimicrobial-resistant *Escherichia coli* (*E. coli*) circulating across the human-animal-environmental interface is a major one-health concern. This study (April 2025–January 2026) investigated the occurrence, virulence, and antimicrobial resistance (AMR) patterns of *E. coli* across 558 samples comprising animal (*n* = 444), environmental (*n* = 80), and human (*n* = 34) sources collected from five organized dairy farms representing urban (*n* = 1), peri-urban (*n* = 2), and rural (*n* = 2) settings under a One Health framework. Samples were cultured for *E. coli* and *E. coli O157*, screened for Shiga-toxin genes (*stx1*, *stx2*) by polymerase chain reaction (PCR), and subjected to antimicrobial susceptibility testing and molecular detection of resistant genes. Overall, *E. coli* was detected in 70.06% of the samples, with the highest occurrence in environmental samples (78.75%), followed by animal (70.94%) and human (47.05%) samples, indicating a clear environmental predominance. In contrast, *E. coli O157* was not detected in any of the samples. Virulence profiling revealed the presence of Shiga-toxin encoding genes *stx1* (10.1%) and *stx2* (13.7%), predominantly among animal isolates, with higher occurrence during monsoon and winter seasons. Antimicrobial susceptibility testing revealed high susceptibility to colistin (100%), tigecycline (97.2%), and carbapenems (>91%), while moderate resistance was observed against tetracycline (25.1%) and selected cephalosporins. Multidrug resistance (MDR) was identified in 18.02% of isolates, with an overall MAR index of 0.068. Molecular analysis showed a predominance of *AmpC* (33.25%), followed by *tetA* (20.05%), *blaTEM* (15.48%), *blaCTX-M* (12.18%), *tetB* (9.39%), and *blaNDM* (2.28%) genes, with notable seasonal variation. These findings indicate that the livestock ecosystem acts as a reservoir for resistant and potentially virulent *E. coli*, underscoring the need for integrated One Health AMR surveillance and targeted mitigation strategies.

## 1 Introduction

Milk is a highly nutritious food essential for new-born mammals, including humans, providing immunity and vital nutrients required for growth and development (1, 2). India is the leading global producer of milk, contributing approximately 25% of total production, with an annual output of 239.3 million tonnes and a per capita availability of 471 grams per day (3, 4). Increasing population, changing lifestyles, and greater dependence on dairy as a major protein source further contributes to increasing milk consumption, particularly in developing countries (1).

However, unlike developed nations, where only a small proportion of milk is consumed raw, a significant portion in developing countries is consumed unpasteurized due to perceived health benefits (5–7). This practice, combined with poor farm-to-table hygiene, increases the risk of microbial contamination, making raw milk a potential vehicle for zoonotic food-borne pathogens (8). Accurate detection of these pathogens in contaminated food materials is therefore essential for effective food safety surveillance, with conventional culture-based methods complemented by molecular, immunological, MALDI-TOF-MS, and next-generation sequencing approaches (9).

Among these, *E. coli* is of particular concern due to its dual role as a commensal and a pathogen responsible for food-borne and opportunistic infections (10, 11). Pathogenic *E. coli* strains are broadly classified based on their virulence mechanisms and the clinical syndromes they produce, including Shiga toxin-producing *E. coli* (STEC) which encompasses the enterohemorrhagic *E. coli* (EHEC) subtype—along with enterotoxigenic *E. coli* (ETEC), enteropathogenic *E. coli* (EPEC), enteroinvasive *E. coli* (EIEC), enteroaggregative *E. coli* (EAggEC), and uropathogenic *E. coli* (UPEC), the latter causing urinary tract infections in both humans and animals (12, 13).

In particular, STEC, are among the most studied food-borne zoonotic pathogens, with cattle serving as the primary reservoirs. STEC strains produce Shiga toxins encoded by *stx1* and *stx2* genes, which are associated with severe clinical conditions such as diarrhea, haemorrhagic colitis, and haemolytic uraemic syndrome (14, 15). Recent studies have further reported virulence-associated genes and AMR in *E. coli* obtained from dairy products and animal-associated sources, highlighting their continued food-safety and public-health significance (16, 17). Transmission to humans primarily occurs through contaminated food and water, direct contact with infected animals, or person-to-person contact (14, 18). Previous studies also reported the fecal shedding of STEC, particularly serotype O157, frequently in young calves immediately after weaning, highlighting the importance of healthy cattle in the epidemiology of transmitting potentially pathogenic *E. coli* (19, 20).

In addition, the rising burden of AMR has emerged as a major global public health challenge, particularly in developing countries (10). Factors such as socio-economic status, low literacy rates among livestock owners, inadequate water, sanitation, and hygiene (WASH) infrastructure, lack of proper prevention and control (IPC) measures, and indiscriminate use of antimicrobials among livestock populations serving livestock farms as hotspots for the emergence and spread of MDR within livestock ecosystems (21, 22). Recognizing their clinical and public health significance, the World Health Organization has classified third-generation cephalosporin-resistant Enterobacterales and carbapenem-resistant Enterobacterales among critical-priority pathogens, underscoring the serious public health threat posed by antimicrobial-resistant *E. coli* belonging to these resistance categories (23).

The inappropriate use of antimicrobials in humans and animals can exert selective pressure favoring resistant organisms and may contribute to the dissemination of antibiotic resistance genes (ARGs) (24). A significant proportion of administered antibiotics was excreted unmetabolized, contaminating the environment via manure, slurry, and wastewater, leading to contamination of ground and surface waters with antibiotic residues, antibiotic-resistant bacteria (ARB), and ARGs (25–27). Furthermore, *E. coli* serves as an important indicator of fecal contamination and commonly harbors extended-spectrum β-lactamase (ESBL) genes, which confer resistance to several β-lactam antibiotics and facilitate the spread of resistance among bacterial populations (28–30).

Several studies from India have reported antimicrobial-resistant *E. coli* in food-producing animals and their environment (31–33), however, these investigations have generally focused on specific components of the farm ecosystem. For example, Jindal et al. (34) characterized phenotypic and genotypic AMR among *E. coli* and *Klebsiella* from dairy farm milk, slurry, and water samples in Punjab, while Yadav et al. (35) investigated ESBL-positive *E. coli* and their resistance profiles in cattle from eastern Uttar Pradesh. More recently, Vijay et al. (36) assessed *E. coli* in milk, animal handlers, and slurry from dairy herds in Punjab, and Narwal et al. (37) investigated molecular characterization of resistant *E. coli* from dairy farm environments. Although these studies have substantially expanded the understanding of AMR in Indian dairy settings, across diverse sample sources, the interconnectedness of AMR among the different components of the farm ecosystem remain poorly characterized. In particular, there is a paucity of data that simultaneously evaluate human, animal, and environmental components within the same organized dairy farms while incorporating temporal variation and both phenotypic and molecular characteristics of *E. coli*.

This represents an important knowledge gap, as AMR and pathogen dissemination within dairy farms are not confined to a single reservoir but may involve interactions among interconnected farm compartments and may vary over time and across seasons. Moreover, studies restricted to a single compartment or a cross-sectional study design may not fully understand this complexity. A One Health approach integrating multiple compartments with repeated sampling over time is essential to understand the true dynamics of *E. coli*, its virulence, and AMR within dairy farm ecosystems.

Against this background, the present study was undertaken as a longitudinal investigation of *E. coli*, *E. coli O157*, their virulence, AMR, and MDR across five organized dairy farms representing urban, peri-urban, and rural settings in Nagpur district, Maharashtra, India. This investigation encompassed human, animal, and environmental components of the livestock ecosystem, and further enabled evaluation of seasonal variation and farm-level factors associated with pathogen occurrence, virulence, and resistance. By examining these interconnected components over time, this study provided an integrated understanding of the epidemiology and transmission potential of *E. coli* in dairy farm settings, highlighting the importance of One Health surveillance, antimicrobial stewardship, improved farm-level management practices and coordinated public health strategies for addressing AMR.

## 2 Materials and methods

### 2.1 Study design and sample collection

Five organized dairy farms representing urban (*n* = 1), peri-urban (*n* = 2), and rural (*n* = 2) settings in Nagpur district, were selected for the study (Figure 1). A total of 558 samples, comprising milk (*n* = 217), dairy cattle fecal samples (*n* = 227), human stool samples (*n* = 34), and environmental samples (*n* = 80) including manure, sewage, feed, and water (*n* = 20 each) were collected aseptically (Figure 2). The study followed a longitudinal design, with animal and environmental samples collected at two-month intervals over a period of 1 year, while human stool samples were collected quarterly. All the samples were collected using sterile containers and were transported to the laboratory, while maintaining the cold chain and were processed using standard microbiological protocols (13).

**Figure 1:**
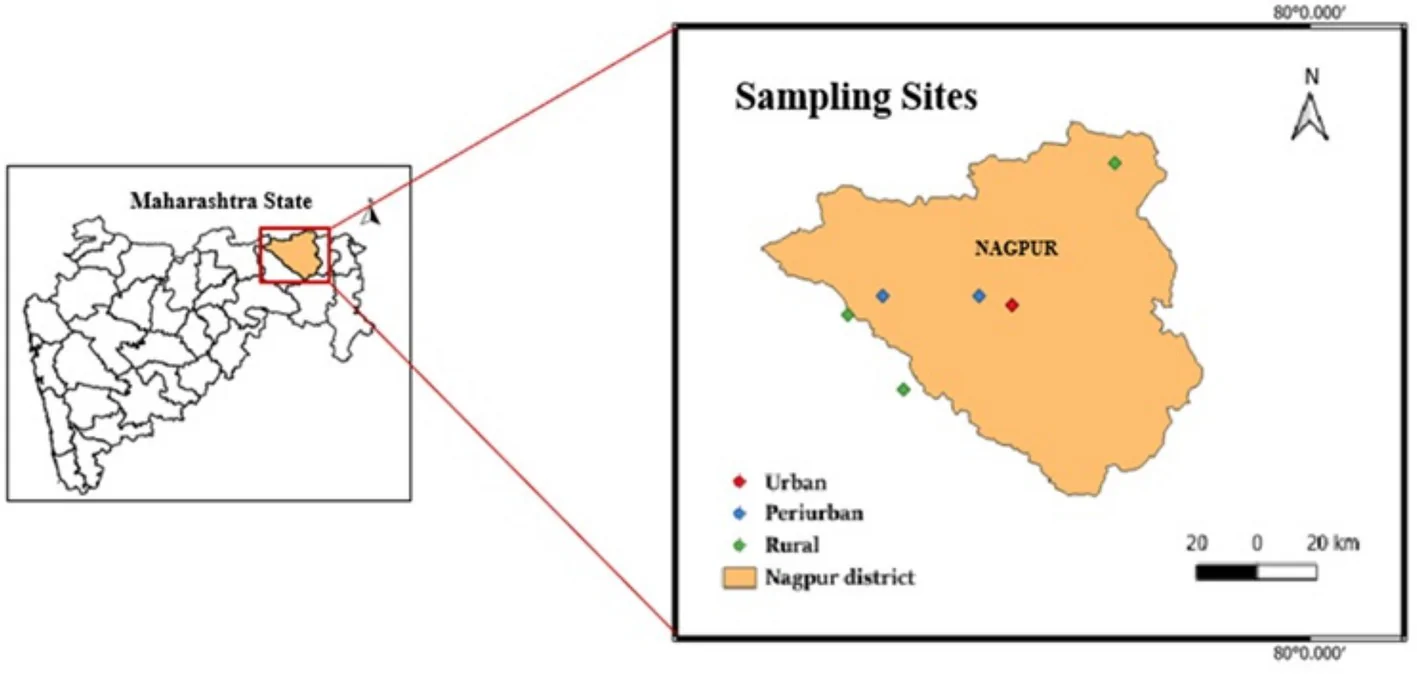
Study area map illustrating sampling locations of livestock farms in Nagpur district.

**Figure 2:**
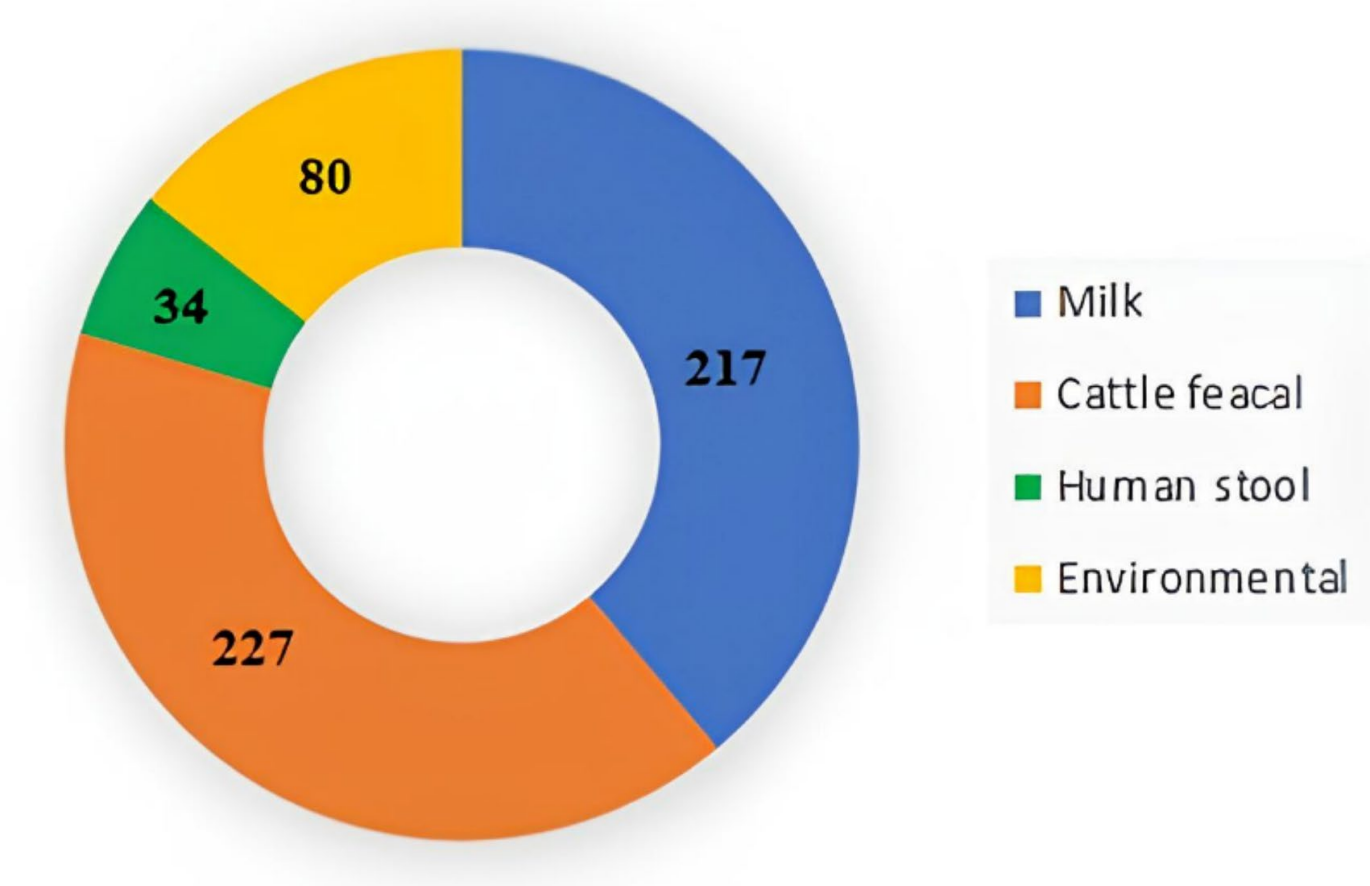
Distribution of samples out of total (*n* = 558).

### 2.2 Processing of the samples

All the samples were then processed under aseptic conditions for bacteriological analysis using standard protocols. Approximately 25 g of feces, manure, and feed samples were collected and homogenized using Phosphate Buffered Saline (PBS) at a 1:10 ratio. On the other hand, milk and sewage samples were mixed by gentle inversion, while drinking water samples were subjected to a ten-fold serial dilution before pre-enrichment.

### 2.3 Isolation and identification of *E. coli* and *E. coli O157*

Following sample preparation, enrichment was carried out in MacConkey broth and incubated at 37 °C for 18–24 h. A loopful of the enriched culture was streaked onto HiCrome *E. coli* Agar and incubated at 37 °C for 24–48 h. Colonies measuring 1–2.5 mm with a characteristic bluish-green colouration were presumptively identified as *E. coli.* Similarly, samples were enriched in Buffered peptone water supplemented with vancomycin, cefixime and cefsulodin for the isolation of *E. coli O157*, followed by streaking onto selective sorbitol MacConkey agar. Non-sorbitol fermenting colonies were further streaked onto Levine Eosin Methylene Blue agar (EMB) and incubated overnight at 37 °C. Colonies exhibiting a characteristic blue-black with a metallic sheen were presumptively identified as *E. coli O157:H7.* All these presumptive isolates were then subjected to Gram staining for further confirmation.

### 2.4 Biochemical characterization of the isolates

All the presumptive isolates were characterized using standard biochemical tests, such as catalase, oxidase, indole, methyl red (MR), Voges-Proskauer (VP), citrate utilization, triple sugar iron test (TSI) and sugar fermentation tests, according to the protocol described by Agarwal et al. (38).

### 2.5 Identification by MALDI-TOF MS

The presumptive bacterial isolates were identified using Matrix-Assisted Laser Desorption Ionization Time-of-Flight Mass Spectrometry (MALDI-TOF MS). Pure colonies grown on BHI agar were spotted onto target slides, overlaid with CHCA (α-cyano-4-hydroxycinnamic acid) matrix solution, and air-dried before the analysis. A reference *E. coli* strain (ATCC 8739) was used for calibration and quality control. The generated spectral data were analysed using the Saramis database (version 4.0; bioMérieux), and identifications with confidence levels between 70 and 99.9% were considered reliable at the genus and species levels.

### 2.6 Molecular confirmation of the isolates by polymerase chain reaction (PCR)

All the presumptive *E. coli* isolates were further subjected to molecular analysis targeting the species-specific *uidA* gene and *rfbE* gene, encoding for *O157* antigen production. Confirmed isolates were further screened for key virulence genes *stx1* and *stx2*, associated with shiga toxin production. The details of the primers and their cycling conditions are mentioned in Supplementary Table S1 (89–91).

Genomic DNA was extracted using the snap-chill method. Initially, 1 mL of the overnight incubated BHI broth cultures was centrifuged at 15,000 rpm for 15 min and washed repeatedly with 500 μL of PBS. The resulting pellet was resuspended in 200 μL of nuclease-free water, heat-treated at 95 °C for 20 min, and immediately snap-chilled at −20 °C for 15 min. The resultant supernatant was removed and used as a DNA template.

PCR was performed in a 25 μL reaction mixture containing 12.5 μL of 2X Go Taq master mix, 8.5 μL of nuclease-free water, 1 μL each of forward and reverse primers (10 pmol/μL), and 2 μL of DNA template and then subjected to amplification following the cycling conditions mentioned in Supplementary Table S1. The resultant PCR products were analysed using the agarose (1.5%) gel electrophoresis (100 V cm^−1^, 20 min and 80 mA) in 1X TAE buffer (pH 8.0), using horizontal gel assembly by adding 0.5 μg/mL of ethidium bromide and visualized under UV illuminator to detect the amplified products.

### 2.7 Antimicrobial susceptibility testing

#### 2.7.1 Kirby-Bauer disk diffusion method

Antimicrobial susceptibility of the confirmed isolates was determined using the Kirby-Bauer disk diffusion method with a panel of commonly used antibiotics, following standard protocols (39). The antibiotic disks used for *E. coli* were Amoxycillin/clavulanic acid (30 μg), Piperacillin/Tazobactam (100/10 μg), Cefuroxime (30 μg), Ceftazidime (30 μg), Cefepime (30 μg), Ceftriaxone (30 μg), Ciprofloxacin (5 μg), Amikacin (30 μg), Tigecycline (15 μg), Tetracycline (30 μg), Gentamicin (10 μg), Fosfomycin (200 μg), Co-Trimoxazole (25 μg), Imipenem (10 μg), Meropenem (10 μg), Ertapenem (10 μg), and Colistin sulfate (10 μg).

Mueller-Hinton agar plates were inoculated with the overnight broth cultures and were incubated at 37 °C for 18–24 h with antibiotic disks placed aseptically, and inhibition zones were measured and interpreted as Sensitive (S), Intermediate (I), or Resistant (R) according to CLSI (Clinical and Laboratory Standards Institute) guidelines.

#### 2.7.2 VITEK 2 analysis

Representative isolates that tested positive for the presence of virulence genes were further analysed using the VITEK 2 system while considering the resource requirements of the system (40). Purified cultures were adjusted to a 0.5 McFarland standard by suspending in 0.45% sterile saline and processed using AST cards. Results were interpreted as Sensitive (S), Intermediate (I), or Resistant (R) according to CLSI guidelines.

#### 2.7.3 Multiple antibiotic resistance (MAR) index

The MAR index was calculated using the formula described by Krumperman (41).


MARindex=a/b

where:

- *a* = number of antibiotics to which the isolate was resistant
- *b* = total number of antibiotics tested against that isolate

### 2.8 Detection of extended spectrum β-lactamases (ESBLs) production

All the isolates showing an inhibition zone of <27 mm for cefotaxime and <25 mm for ceftriaxone were screened for ESBL production. Confirmation was done using the Modified Double Disc Synergy Test (MDDST), where a uniform lawn culture of the test organisms was prepared on Mueller–Hinton agar plates, with an amoxicillin-clavulanate disk placed at the centre, and third-generation (cefotaxime, ceftriaxone, and cefpodoxime) and fourth-generation (cefepime) cephalosporins were positioned at distances of 15 mm and 20 mm, respectively. An increase or distortion of the inhibition zone toward the amoxicillin-clavulanate disk indicated ESBL production (42).

### 2.9 Detection of antimicrobial resistance (AMR) genes

All phenotypically confirmed *E. coli* isolates were screened for AMR genes targeting *tetA* and *tetB* (tetracycline resistance), associated with tetracycline, *blaTEM* and *blaCTX-M* variants (group 1, group 2 and group 9) for ESBL production, *blaNDM* for carbapenem resistance, along with plasmid-mediated *AmpC* genes. PCR products were analysed by agarose gel electrophoresis, and primer details and cycling conditions are provided in Supplementary Table S2.

### 2.10 Data analysis

All the data were entered in Microsoft Excel and analysed using appropriate statistical software. Associations between independent variables such as sample type, season, farm characteristics, milking method, water source, and hygiene practices and outcome variables (*E. coli* occurrence, virulence profiles, and MDR) were analysed using three separate multivariable binary logistic regression models. For each model, the outcome variable was coded as binary (1 = presence and 0 = absence). Adjusted odds ratio (ORs) with 95% confidence intervals (CIs) were calculated from the regression coefficients. Multicollinearity among the independent variables was assessed using variation inflation factors (VIFs), with all the values below 2.2, indicating no evidence of problematic collinearity. The overall significance of each model was assessed using the likelihood-ratio, chi-square test, and model performance was summarized using McFadden’s pseudo-R (38).

The discriminatory ability of the models was evaluated using the area under the receiver operating characteristic curve (AUC), while model calibration was done using the Hosmer–Lemeshow goodness-of-fit test and Brier score. All tests were two sided and a *p*-value <0.05 were considered statistically significant.

## 3 Results

### 3.1 Isolation and identification of *E. coli* and *E. coli O157*

All collected samples were processed immediately to isolate *E. coli* and *E. coli* O157, following standard protocols as described in Section 2.4. Out of 558 samples analysed, 394 (70.06%) were presumptively identified as *E. coli*, including 315/444 animal, 63/80 environmental, and 16/34 human samples, based on characteristic bluish-green colonies on HiCrome *E. coli* agar (Supplementary Figure S1). Additionally, 88 isolates (71 animal, 10 environmental, and 7–human) were presumptively identified as *E. coli* O157 by non-sorbitol fermentation and a characteristic metallic sheen appearance on EMB agar (Supplementary Figure S2). All isolates were confirmed as Gram-negative bacilli (Supplementary Figure S3) upon microscopic examination.

### 3.2 Biochemical characterization

All 394 presumptive *E. coli* isolates were subjected to biochemical tests, where 100% (394/394) were positive for catalase and negative for oxidase tests. The majority were positive for indole (99.2%; 391/394), methyl red (97.4%; 384/394), and triple sugar iron (97.9%; 386/394) tests, while 96.7% (381/394) and 96.1% (379/394) were negative for Voges-Proskauer and citrate tests, respectively. Additionally, 97.7% (385/394) fermented glucose, while 96.9% (382/394) did not ferment sucrose, confirming the typical biochemical profile of the *E. coli*.

Similarly, among 88 presumptive *E. coli O157* isolates, all were catalase positive and oxidase negative. Most isolates were positive for indole (95.4%; 84/88), methyl red (92.04%; 81/88) and triple sugar iron test (94.3%; 83/88), while 90.9% (80/88) were negative for Voges-Proskauer and were able to ferment glucose. Additionally, citrate utilization was absent in 88.6% (78/88) of the isolates, and sucrose fermentation was not observed in 89.7% (79/88) of the isolates.

Following biochemical characterisation, despite variation in test results, all 394 isolates were subjected to molecular analysis, as molecular methods are considered more reliable than biochemical tests due to inconsistencies in phenotypic identification observed in our earlier studies.

### 3.3 Molecular confirmation and occurrence

All 394 presumptive *E. coli* isolates from animal, human, and environmental samples were confirmed by PCR targeting the species-specific *uidA* gene, a reliable marker encoding β-glucuronidase activity, which produced a 630 bp amplicon (Supplementary Figure S4). However, none of the 88 presumptive *E. coli O157* isolates were positive for the *rfbE* gene, indicating that *E. coli O157* was not detected in the samples collected during the study period.

The overall occurrence of *E. coli* was highest in environmental samples (78.75%), followed by animal (70.94%), and human samples (47.05%) as depicted in Figure 3. Seasonal analysis revealed distinct temporal trends in the occurrence of *E. coli* across different sample types, where animal samples showed relatively stable occurrence (67.5–74.7%) throughout the study period, while environmental samples exhibited marked seasonal variation, increasing from 55% during summer (first collection) to 95% during the peak monsoon season (third collection), followed by a slight decline to 85% during winter (fourth collection). Human samples showed comparatively lower and more variable occurrence (42.8–60%; Figure 4).

**Figure 3:**
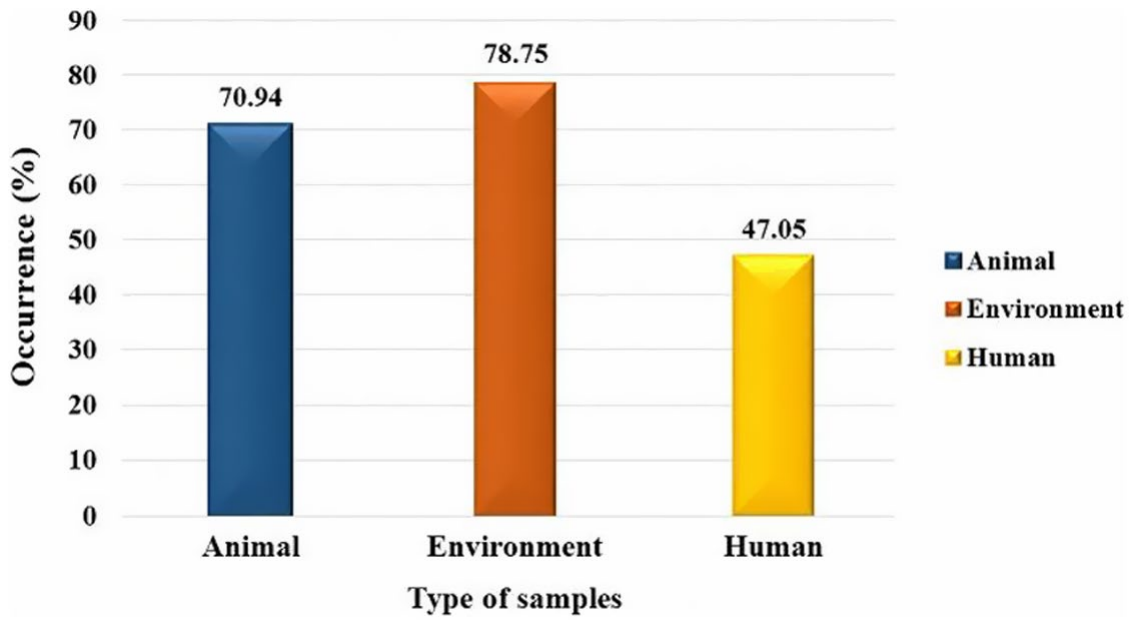
Overall occurrence of *E. coli* isolates across five dairy farms.

**Figure 4:**
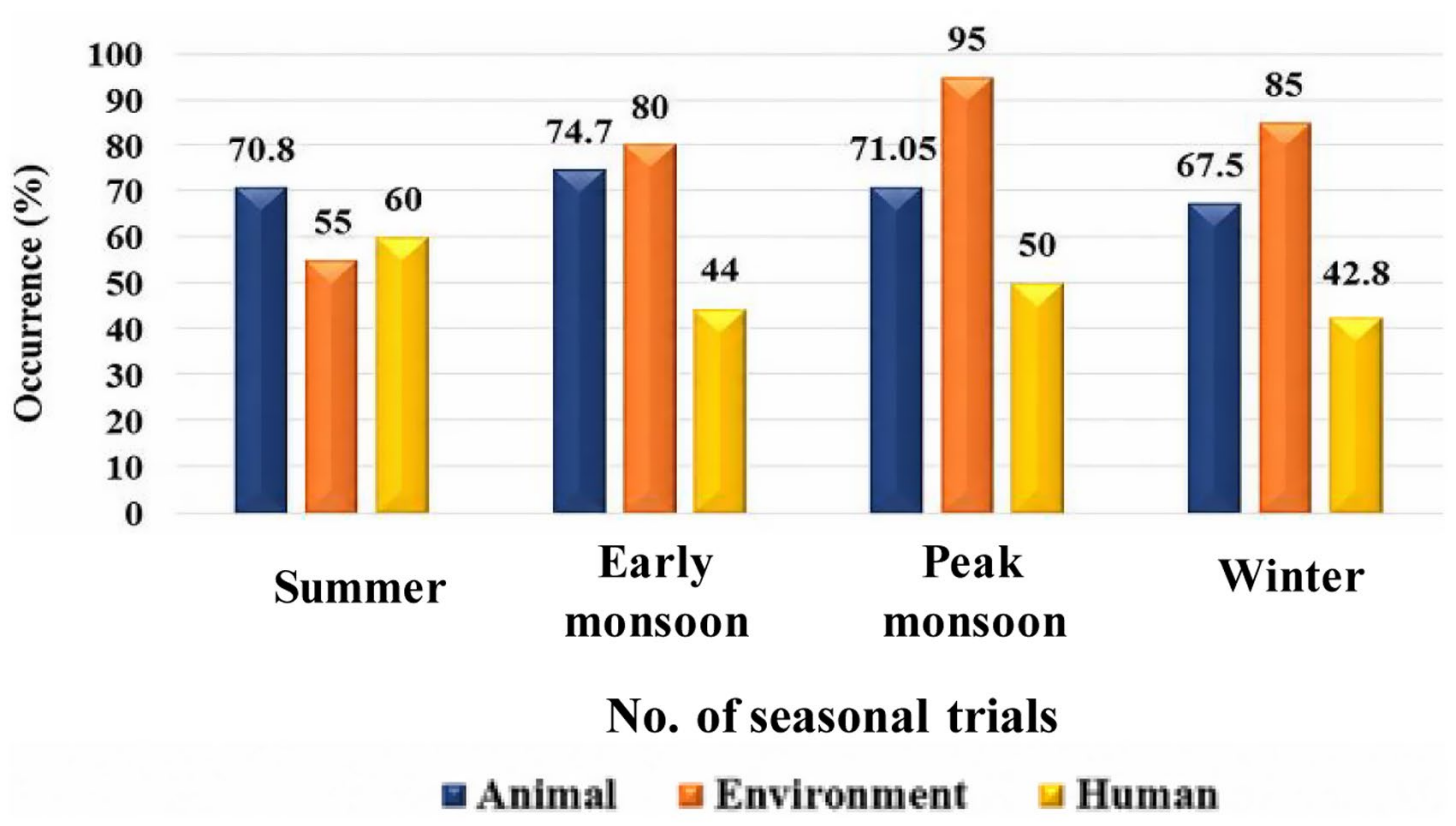
Seasonal occurrence of *E. coli* isolates across four sample collections from five livestock-associated dairy farms.

### 3.4 Identification by MALDI-TOF-MS

MALDI-TOF MS is a rapid and sensitive technique for microbial identification based on protein spectral analysis. Among 96 representative isolates analysed, 83.3% (80/96) were correctly identified as *E. coli* at the species level, while eight isolates were misidentified as *Enterococcus faecalis* and eight remained unidentified.

### 3.5 Detection of virulent genes

All the molecularly confirmed *E. coli* isolates (*n* = 394) were screened for virulence genes *stx1* and *stx2* to assess pathogenic potential. Among 394 *E. coli* isolates, 10.1% (40/394) were positive for *stx1,* producing an amplicon of 130 bp (Supplementary Figure S5A) and 13.7% (54/394) for *stx2*, with an amplicon of 346 bp (Supplementary Figure S5B), indicating the higher occurrence of the major virulent strain (*stx2*). Higher occurrence was observed in animal samples (*stx2*: 15.2%; *stx1*: 11.7%) compared to environmental samples (*stx2*: 9.5%; *stx1*: 4.7%), while none of the human samples tested positive (Figure 5).

**Figure 5:**
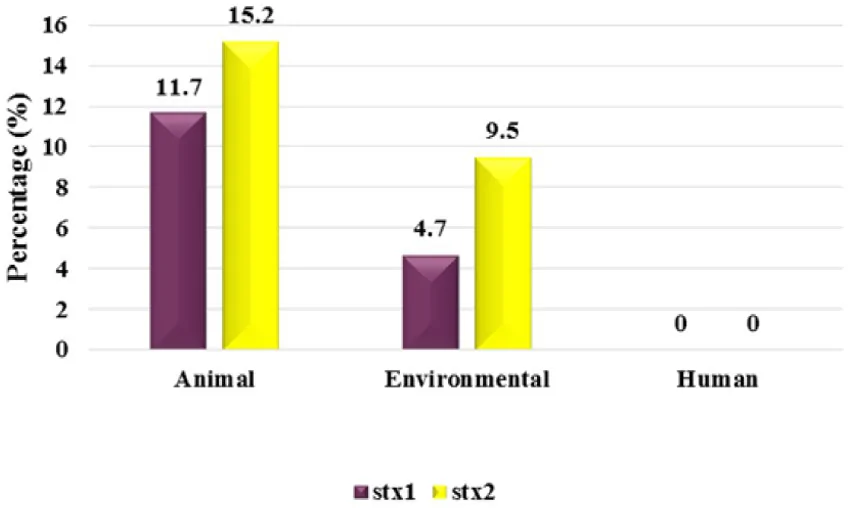
Overall occurrence of *stx1* and *stx2* virulent genes of *E. coli*.

Seasonal analysis revealed temporal variation, with *stx1* (9.19%) predominating over *stx2* (5.74%) during summer (first collection). During early monsoon (second collection), both the genes exhibited a higher occurrence of 14% (*stx2*) and 13% (*stx1*). A similar trend was observed during peak monsoon (third collection), with the occurrence of *stx1* and *stx2* in 12.6 and 11.6% of the isolates, respectively. In contrast, a marked increase was noticed in the occurrence of *stx2* (22.11%), while *stx1* was detected only in 5.76% of the isolates during winter (fourth collection; Figure 6).

**Figure 6:**
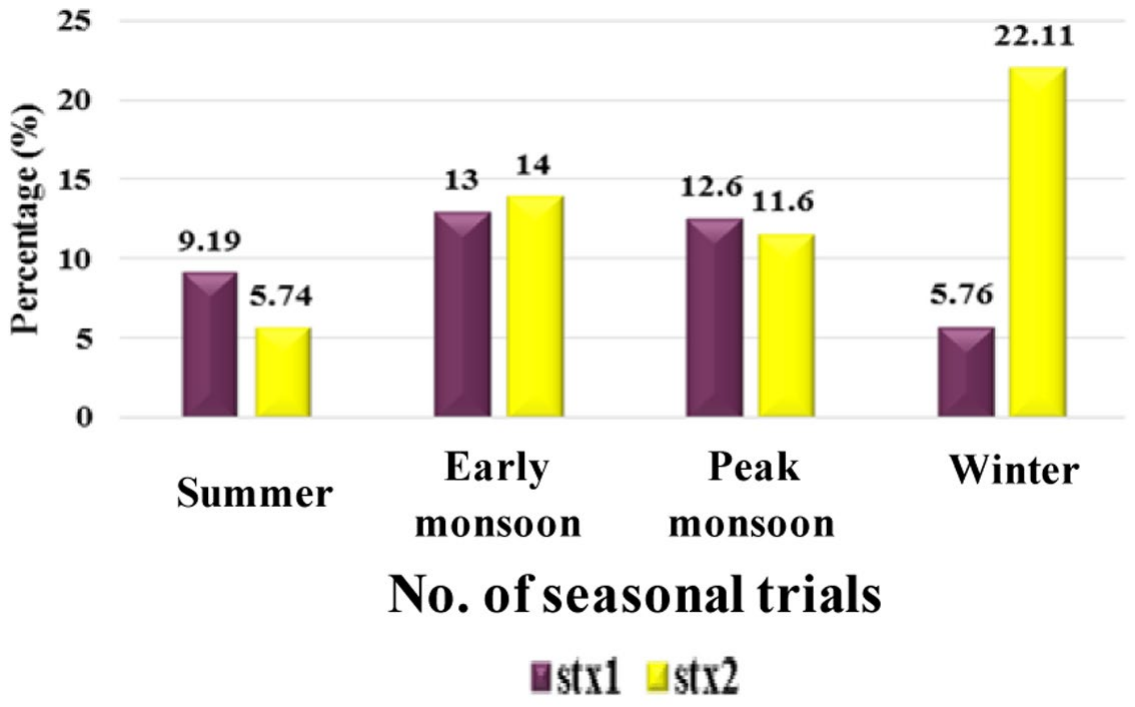
Seasonal occurrence of *stx1* and *stx2* virulence genes of *E. coli* across four sample collections.

### 3.6 Antimicrobial susceptibility profile

#### 3.6.1 Kirby-Bauer disk diffusion method

All 394 confirmed *E. coli* isolates were analysed for antimicrobial susceptibility using the Kirby-Bauer disk diffusion method against 17 antibiotics, revealing varied resistance and sensitivity patterns (Supplementary Table S3).

High sensitivity was observed for colistin sulfate (100%), tigecycline and ertapenem (97.2%), cotrimoxazole (96.4%), meropenem (94.6%), and imipenem (91.8%), followed by cephalosporins and other antibiotics (Figure 7). Based on the observations, the MAR index was calculated to assess the antimicrobial exposure within the targeted livestock ecosystem. Of the 17 antibiotics, the MAR index was highest for tetracycline (0.251), indicating its frequent use, followed by ceftriaxone (0.162), cefuroxime (0.155), ciprofloxacin (0.102), and amikacin (0.102). In contrast, most other antibiotics, such as ceftazidime, cefepime, cotrimoxazole, tigecycline, piperacillin-tazobactam, gentamicin, and carbapenems, showed low MAR indices (<0.1), indicating limited use in the studied livestock settings.

**Figure 7:**
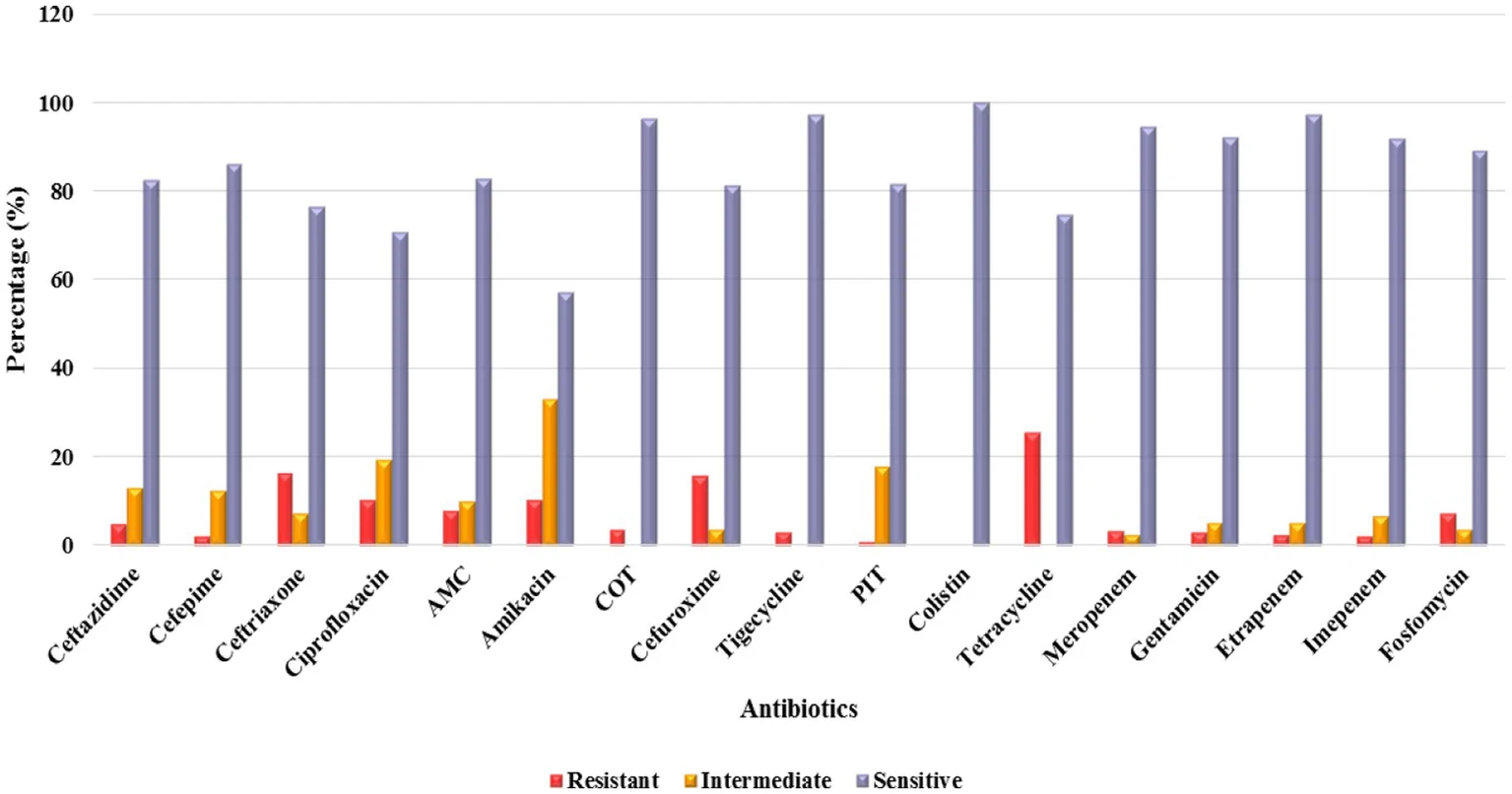
Antibiogram profile of *E. coli* isolates.

Seasonal analysis demonstrated a gradual increase in the resistance patterns from summer to winter. The lowest MAR index was recorded during summer (0.014), where most isolates exhibited high susceptibility to the tested antibiotics (Supplementary Figure S6). Resistance gradually increased during early monsoon (0.063) and peak monsoon (0.083), particularly against tetracycline, ciprofloxacin, and ceftazidime, as depicted in Supplementary Figures S7, S8, respectively. The highest resistance burden was observed during winter, with a MAR index of 0.106, characterized by reduced susceptibility and increased MDR among isolates (Supplementary Figure S9), indicating enhanced circulation and persistence of resistant *E. coli* strains during monsoon and winter periods. The seasonal resistance profile of *E. coli* isolates against the tested antibiotics is presented as a heat map (Figure 8), with values representing the percentage of resistant isolates across the four sampling seasons.

**Figure 8:**
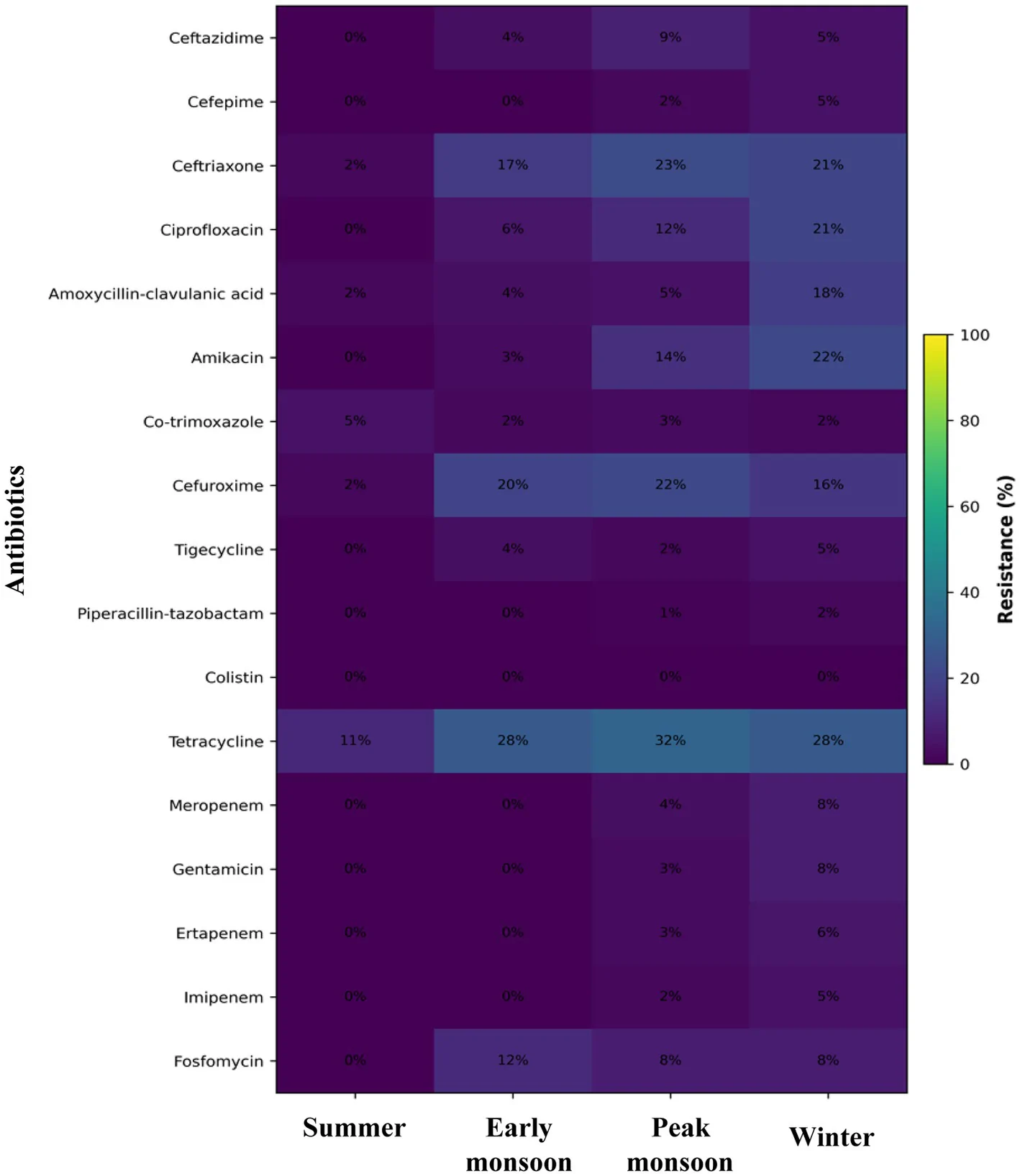
Seasonal resistance profiles of *E. coli* isolates.

#### 3.6.2 VITEK 2 analysis

A total of 25 representative *E. coli* isolates, harboring virulence genes, were further analysed using the VITEK 2 system. Among these, five isolates exhibited resistance to cephalosporins, four to colistin, and two to minocycline and tigecycline. The remaining isolates were susceptible to other tested antibiotics. The susceptibility patterns obtained using VITEK 2 did not completely align with those observed by the Kirby–Bauer disk diffusion method in particular to colistin. These differences may be attributed to the methodological differences, antibiotic diffusion characteristics, and interpretative criteria. In particular, disk diffusion is not considered a reliable method for determining colistin susceptibility because of its poor diffusion in agar media; therefore, broth microdilution or automated systems such as VITEK 2 are recommended for colistin susceptibility testing according to CLSI and EUCAST guidelines (43). The Kirby–Bauer disk diffusion method was used for susceptibility profiling of all 394 confirmed *E. coli* isolates, whereas VITEK 2 analysis was performed on 25 representative virulence-gene-positive isolates as an additional susceptibility assessment. Thus, the differences observed between the two methods should be interpreted in the context of their different testing approaches and isolate subsets.

### 3.7 ESBLs production

The modified double disk synergy test (MDDST) was employed to detect ESBL production among *E. coli* isolates obtained from the livestock settings. Representative MDR isolates, particularly those with reduced susceptibility to third and fourth-generation cephalosporins, were screened, and nearly all the tested isolates (100%) were identified as ESBL producers.

### 3.8 Detection of AMR genes

All 394 molecularly confirmed *E. coli* isolates were screened for AMR genes, targeting *tetA, tetB, blaTEM*, *blaCTX-M* variants, *blaNDM*, and *AmpC* genes as described in section 2.10. Among these, 20.05% (79/394) and 9.39% (37/394) were positive for the *tetA* (502 bp) and *tetB* (930 bp) genes, respectively (Supplementary Figures S10A,B). Among the ESBL-associated genes, *blaTEM* (800 bp) was detected in 15.48% (61/394) of the isolates (Supplementary Figure S10C) while *blaCTX-M* variants were identified in 12.18% (48/394) of the isolates, with amplicon sizes of 688 bp (group 1), 404 bp (group 2), and 561 bp (group 9) as shown in the Supplementary Figure S10D. Additionally, 2.28% (9/394) of the isolates carried the *blaNDM* gene (475 bp; Supplementary Figure S10E), while 33.25% (131/394) harbored the *AmpC* gene (634 bp; Supplementary Figure S10F), making it the most prevalent resistance gene as illustrated in Figure 9.

**Figure 9:**
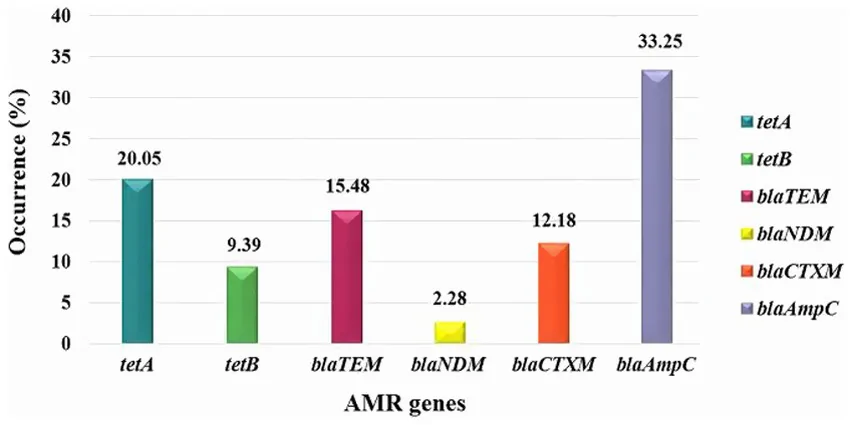
Overall occurrence of AMR genes in *E. coli* isolates.

The distribution of AMR genes among *E. coli* isolates varied across different sample types within the livestock ecosystem. Tetracycline resistance genes *tetA* (26.9%) and *tetB* (11.11%) were more prevalent among the environmental samples compared to animal (*tetA* 19.68%; *tetB* 9.52%) samples, while none of the human samples tested positive. Among ESBL genes, *blaTEM* showed the highest occurrence among the human isolates (25%), followed by environmental (19.04%) and animal isolates (14.28%). A similar trend was observed for *blaCTX-M*, with higher detection in environmental (19.04%) and human (18.75%) isolates compared to animal isolates (10.47%). The plasmid-associated *AmpC* gene was the most predominant across all sample sources, with the highest occurrence in human (62.5%), followed by environmental (46.03%) and animal isolates (29.2%). In contrast, the carbapenem resistance gene *blaNDM* was detected at lower frequencies but was higher in human isolates (12.5%) compared to environmental (7.6%) and animal isolates (1.26%). Overall, these findings indicate the circulation of key AMR genes within the livestock ecosystem, with the environment and humans acting as important reservoirs, highlighting the risk of cross-sectoral transmission (Figure 10).

**Figure 10:**
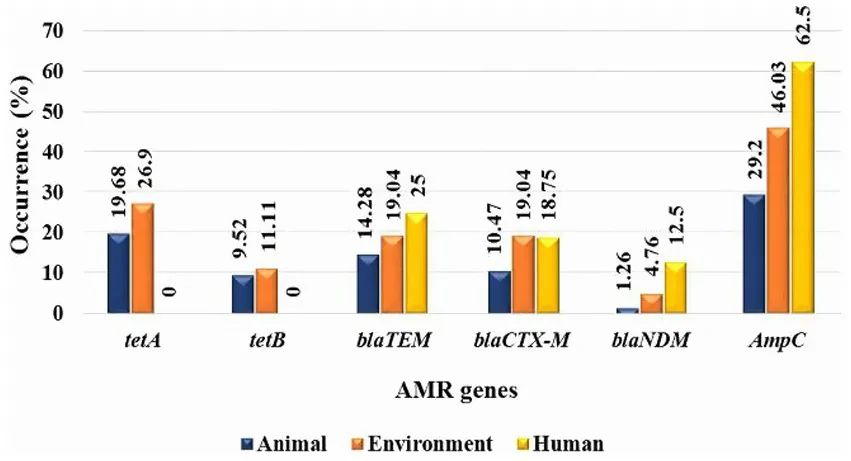
Overall occurrence of AMR genes in *E. coli* isolates across different sample types.

This study demonstrated a clear seasonal variation in the occurrence of resistance genes during the study period. The resistance burden increased progressively from summer to winter, with lower detection noticed during summer, with the occurrence of *tetA*, *blaTEM* and AmpC, *blaCTX-M*, and *tetB* genes in 12.3, 6.84, 5.47, and 2.73% of the animal isolates, respectively. In contrast, the environmental isolates comparatively showed a lower occurrence of the *tetA* (9.09%) AMR gene, while none of the human isolates tested positive (Figure 11). A sharp rise in the occurrence of AMR genes, particularly *AmpC* (62.5% environmental; 50% human; 23.7% animal), was observed during the early monsoon season (Figure 12). Additionally, peak monsoon and winter seasons recorded the highest resistance burden, with 66.6 and 100% of the human isolates harboring the *AmpC* gene, respectively. Similarly, the occurrence of other resistance genes also increased during the peak monsoon and winter seasons, as depicted in Figures 13, 14, respectively.

**Figure 11:**
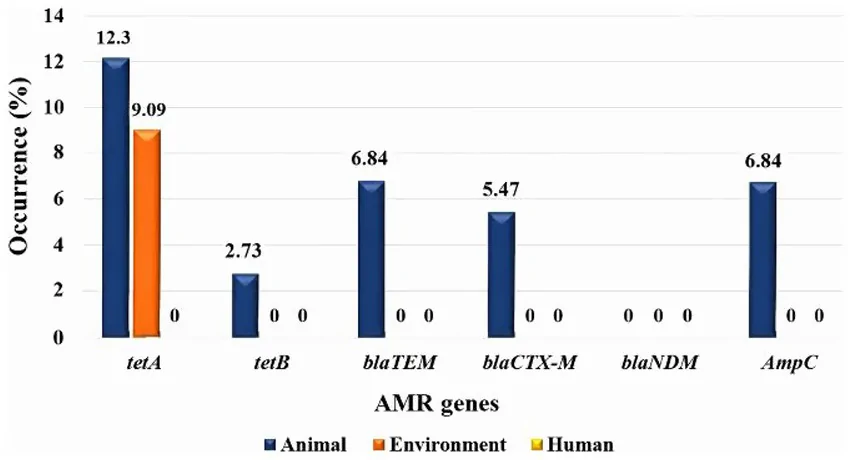
Occurrence of AMR genes in *E. coli* isolates across different sample types during the first collection (summer).

**Figure 12:**
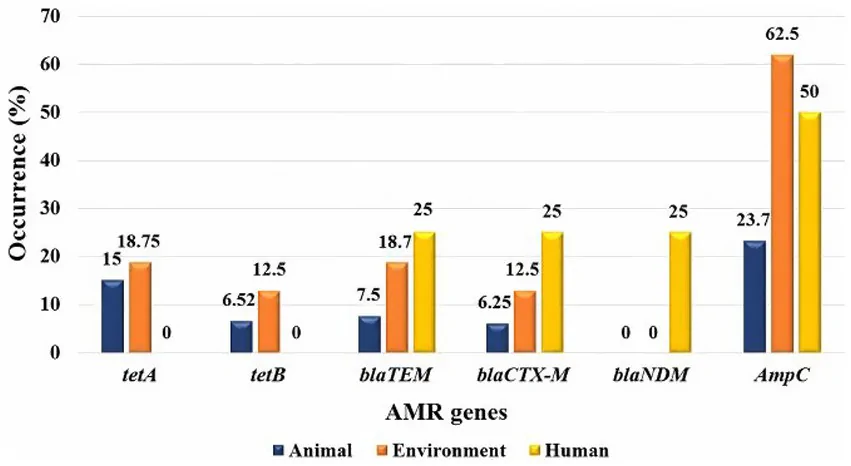
Occurrence of AMR genes in *E. coli* isolates across different sample types during the second collection (early monsoon).

**Figure 13:**
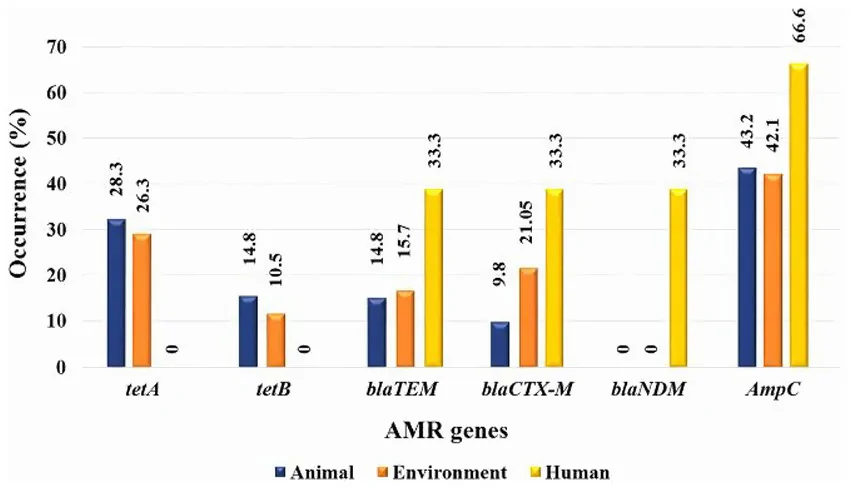
Occurrence of AMR genes in *E. coli* isolates across different sample types during the third collection (peak monsoon).

**Figure 14:**
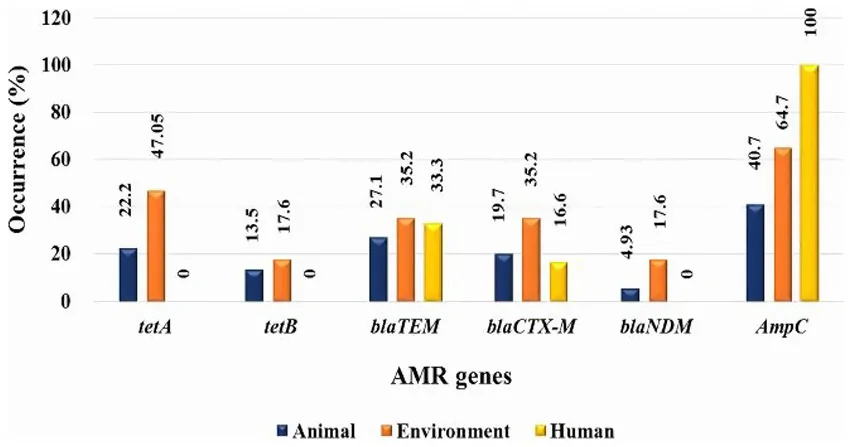
Occurrence of AMR genes in *E. coli* isolates across different sample types during the fourth collection (winter).

Overall, *AmpC* (33.25%) was the predominant AMR gene, followed by *tetA* (20.05%), *blaTEM* (15.48%), *blaCTX-M* (12.18%), and *tetB* (9.39%), while *blaNDM* exhibited the lowest occurrence (2.28%), indicating the seasonal dissemination of AMR genes in the targeted livestock settings.

### 3.9 Multivariable analysis of risk factors associated with *E. coli* occurrence, AMR, and virulence strains

Multivariable regression analysis was performed to assess the association between farm-related risk factors and the occurrence of *E. coli*, virulence strains, and MDR, as presented in Tables 1–3.

**Table 1:** Multivariable logistic regression analysis of risk factors associated with *E. coli* o*cc*urrence.

Variable	Adjusted odd’s ratio (aOR)	95% CI	*p*-value
Sample type	2.56	1.92–3.40	<0.001
Season	1.00	0.83–1.20	0.995
Farm	1.01	0.84–1.22	0.886
Water source	0.88	0.53–1.47	0.628
Milking method	9.17	3.80–22.11	<0.001
Hygiene	0.93	0.54–1.59	0.787

**Table 2:** Multivariable logistic regression analysis of risk factors associated with virulence profiles.

Variable	Adjusted odd’s ratio (aOR)	95% CI	*p*-value
Sample type	1.00	0.76–1.33	0.985
Season	1.36	1.08–1.72	0.009
Farm	1.05	0.76–1.45	0.774
Water source	0.61	0.27–1.39	0.237
Milking method	1.37	0.57–3.30	0.482
Hygiene	0.39	0.18–0.85	0.017

**Table 3:** Multivariable logistic regression analysis of risk factors associated with MDR.

Variable	Adjusted odd’s ratio (aOR)	95% CI	*p*-value
Sample type	1.73	1.31–2.29	<0.001
Season	1.61	1.23–2.10	<0.001
Farm	1.00	0.80–1.26	0.967
Water source	1.43	0.72–2.85	0.314
Milking method	2.63	0.95–7.32	0.064
Hygiene	2.82	1.14–6.96	0.025

For the occurrence of *E. coli*, the overall regression model was statistically significant (*p* < 0.001; McFadden’s pseudo *R*^2^ = 0.146). Among independent variables, sample type (aOR = 2.56, 95% CI: 1.92–3.40, *p* < 0.001) and milking method (aOR = 9.17, 95% CI: 3.80–22.11, *p* < 0.001) were significantly associated with *E. coli* occurrence, whereas season, farm, water source, and hygiene were not statistically significant. The model showed good discriminatory ability (AUC = 0.812). However, the Hosmer–Lemeshow test indicated poor calibration (*p* < 0.001), suggesting that the predicted probabilities did not closely correspond to the observed outcomes and that the model predictions should therefore be interpreted with caution.

For MDR, the regression model was statistically significant (*p* < 0.001; McFadden’s pseudo-*R*^2^ = 0.112). Sample type (McFadden’s pseudo-*R*^2^ = 0.112), and season (aOR = 1.61, 95% CI: 1.23–2.10; *p* < 0.001) were significantly associated, whereas the other variables were found to be not statistically significant. The model demonstrated moderate discriminatory ability, with an apparent AUC of 0.744, indicating relatively stable model discrimination, and the Hosmer–Lemeshow test did not indicate evidence of poor calibration (*p* = 0.222).

Similarly, for virulence profiles, the regression model was statistically significant (*p* = 0.0019) although its explanatory ability was limited (McFadden’s pseudo-*R*^2^ = 0.048). Season was significantly associated with virulence-marker detection (aOR = 1.36, 95% CI: 1.08–1.72; *p* = 0.009), whereas the hygiene category was associated with lower odds of virulence-marker detection (aOR = 0.39, 95% CI: 0.18–0.85; *p* = 0.017). The model showed limited discriminatory ability, with an apparent and cross-validated AUC of 0.666 and 0.624 respectively, indicating limited discrimination. No evidence of poor calibration was detected by the Hosmer–Lemeshow test (*p* = 0.090).

Overall, the multivariable analysis showed that sample type and milking method were significantly associated with *E. coli* occurrence, while sample type, season, and hygiene practices were significantly associated with MDR. Virulence-marker detection was significantly associated with season and hygiene practices. These findings indicate that both farm-related and environmental factors may contribute to the occurrence of *E. coli*, MDR, and virulence-associated strains.

## 4 Discussion

*E. coli* is widely recognized as an indicator of fecal contamination and an important zoonotic pathogen within dairy farm ecosystems, making it highly relevant for One Health-based surveillance. In the present study, a high occurrence of *E. coli* was observed in environmental samples (78.75%), followed by animal (70.94%) and human (47.05%) samples, highlighting the environment as a major reservoir for bacterial dissemination (44). These findings are consistent with earlier reports, including Martins et al. (45), Heijnen and Medema (46), and Sobur et al. (47), as well as studies by Alsanjary and Sheet (48) and Vijay et al. (36), which emphasize the role of environmental sources such as treated water samples, slurry, flies and bedding materials in disseminating zoonotic pathogens.

The high occurrence in animal samples aligns with the findings of Maheux et al. (49), Adesiyun et al. (50), and Yadav et al. (35), while the comparatively lower occurrence in humans is consistent with Awadallah et al. (51) and Vijay et al. (36), though higher detection reported by Narwal et al. (37) underscores the importance of human, animal, and environment interactions.

Notably, *E. coli* O157 was not detected in the present study. Similar occurrence rate or transient detection has been reported by Rahn et al. (52), Sargeant et al. (53), Murinda et al. (54), and Shinde et al. (20), although higher occurrence has been reported by other studies (55, 56). This suggests either a complete absence or low-level circulation of this pathogenic strain in the study area, highlighting the need for continuous surveillance.

This study demonstrated a lower species-level identification rate of *E. coli* by MALDI-TOF MS, with 83.3% (80/96) of the representative isolates correctly identified, compared with higher identification rates reported by Christner et al. (57) (99.6%), and Karademir et al. (58) (100%). The comparatively lower identification rate in the present study may be associated with the heterogenous nature of the isolates, particularly those originating from environmental sources, as well as spectral similarities and limitations in the reference database (59). Variability in MALDI-TOF-MS identification among environmental isolates has also been reported by Suzuki et al. (60). Differences in sample preparation and the availability of representative reference spectra may further influence the quality of protein profiles and identification outcomes. Therefore, the present findings support the use of complementary molecular techniques for confirmation of isolates that are misidentified or remain unidentified by MALDI-TOF-MS.

Similarly, the occurrence of STEC revealed higher detection of *stx2* (13.7%) than *stx1* (10.1%), in the present study, indicating the predominance of more virulent strains. These findings align with those of Fremaux et al. (61) and Ahmed et al. (62), who documented a similar predominance of virulent strains in animal fecal and environmental samples. Higher occurrence in animal samples (*stx2*-15.2%; *stx1*-11.7%) was consistent with the findings of Das et al. (63), Stella et al. (64), and Alam et al. (65), supporting cattle as the primary reservoirs, while comparatively lower occurrence of *stx2*-9.5%; *stx1*-4.7%, among environmental samples was supported by Polifroni et al. (66), suggesting the environment as a secondary reservoir in bacterial dissemination. Complete absence or low level of circulation of these genes in human samples during the study period aligns with the findings of AL-Wahed et al. (67), though occasional detection has been reported by Awadallah et al. (51), suggesting conditional transmission risks.

Antimicrobial susceptibility profiling indicated high sensitivity to last-resort antibiotics such as colistin, tigecycline, carbapenems, and co-trimoxazole, which were consistent with the earlier findings of Shoaib et al. (68), Li et al. (69), and Narwal et al. (37), although lower resistance was also observed, as supported by Veloo et al. (70). In contrast, tetracycline resistance was higher (25.1%), indicating its widespread use, as supported by Fesseha et al. (71), Shoaib et al. (68), Bui et al. (72), and Narwal et al. (37).

Resistance to different classes of cephalosporins observed in this study was comparatively lower than earlier reports (37, 68, 71, 73), but is consistent with the studies documented by Massé et al. (74), Arbab et al. (75), Karademir et al. (58), and Veloo et al. (70), which reported lower resistance levels. Additionally, Aminoglycosides showed variable resistance, with higher susceptibility to gentamicin, aligning with previous studies as reported by Tadesse et al. (73) and Fesseha et al. (71). Recent evidence from dairy-farm environments has also demonstrated the occurrence of MDR *E. coli* carrying diverse resistance, efflux-associated, and virulence genes, highlighting the complexity of AMR within these ecosystems (76). The overall MAR index of 0.068 and MDR prevalence (18.02%) were below the critical threshold (0.2), indicating low antimicrobial pressure, despite a gradual increase in resistance from summer to winter, indicating seasonal and managemental influences (77).

The higher occurrence of AMR and virulence associated characteristics during the monsoon and winter seasons are influenced by the seasonal changes in the farm environment and managemental practices. During the monsoon, increased moisture and humidity as well as cooler conditions during winter may favor bacterial persistence for longer periods in the farm environment such as bedding, water, soil, and other contaminated areas. Seasonal differences may effect the farm environment and managemental practices, influencing the bacterial exposure and selection pressure, thereby contributing to the greater occurrence of resistant and virulent *E. coli* during these seasons. However, these factors were not quantitatively assessed in the present study, therefore should be considered as the possible explanations for the observed seasonal pattern.

Phenotypic screening revealed that nearly all isolates were ESBL producers, similar to the previous studies reported by Kaur et al. (42), Karademir et al. (58), and Narwal et al. (37), indicating widespread β-lactam resistance in the livestock ecosystem. Molecular analysis further confirmed the circulation of AMR genes, with *AmpC* showing the highest occurrence (33.25%), followed by *tetA* (20.05%), *blaTEM* (15.48%), *blaCTX-M* (12.18%), and *tetB* (9.39%), while *blaNDM* was detected at lower levels (2.28%). These findings are supported by Hartmann et al. (78), Borah (79), and Srathongneam et al. (80), although higher prevalence has been reported by Navajas-Benito et al. (81) and Ramaite et al. (82). Tetracycline resistance genes were predominantly detected in animal and environmental isolates, similar to studies reported by Navajas-Benito et al. (81) and Jindal et al. (34), with their increased occurrence during monsoon and winter possibly due to higher antibiotic usage and enhanced environmental persistence (68, 83). The higher occurrence of *blaNDM* in human isolates (12.5%) compared to environmental (4.76%) and animal (1.26%) samples highlights its clinical relevance among the human isolates, as supported by Prendergast et al. (84) and Pumipuntu and Pumipuntu (85).

The higher occurrence of *AmpC* (62.5%) and *blaNDM* (12.5%) among human isolates may be related to the differences in antimicrobial exposure associated with human treatment, particularly the use of β-lactam and broad-spectrum antibiotics. In contrast, lower occurrence observed in animal and environmental isolates further suggests the persistence and circulation of these genes among the interconnected reservoirs. The detection of *blaNDM* is particularly concerning, as it is associated with carbapenem resistance, which can limit the available treatment options. However, the present findings do not provide enough evidence to determine the direction of transmission across different sample sources, emphasizing the need for further genomic surveillance to understand their possible spread across human, animal, and environmental compartments.

Multivariable analysis further identified specific factors associated with *E. coli* occurrence, MDR, and virulence-marker detection. Sample type and milking method were significantly associated with *E. coli* occurrence, while sample type, season, and hygiene practices were significantly associated with MDR. Season and hygiene practices were also significantly associated with virulence-marker detection. The *E. coli* occurrence model showed good discriminatory ability (AUC = 0.812), although its calibration was poor, indicating that the predicted probabilities did not closely correspond to the observed outcomes. In contrast, the MDR model demonstrated moderate and relatively stable discrimination, with an apparent AUC of 0.744 and a cross-validated AUC of 0.718, while no evidence of poor calibration was observed. The virulence model showed limited discriminatory ability, with an apparent AUC of 0.666 and a cross-validated AUC of 0.624, although no significant evidence of poor calibration was detected. The relatively low McFadden’s pseudo-*R*^2^ values, particularly for the MDR (0.112) and virulence (0.048) models, indicate that the variables included in the models explained only a limited proportion of the observed variation. This may reflect the multifactorial nature of *E. coli* occurrence, antimicrobial resistance, and virulence, which may be influenced by several interacting biological, environmental, and management-related factors. Although information on animal health, feed and water sources, hygiene, biosecurity, and farm management was collected through questionnaires, some variables showed limited variation or could not be assessed quantitatively, which may have reduced their ability to explain differences in the outcomes.

In contrast, certain limitations should be considered when interpreting the present findings. The relatively small number of human samples may have limited the generalizability of the human-associated findings. The absence of whole-genome sequencing prevented assessment of genetic relatedness and further confirmation of possible transmission links among animal, human, and environmental isolates. In addition, the lack of quantitative farm-level antibiotic-use data limited assessment of the relationship between antimicrobial exposure and the observed resistance patterns. As the study was restricted to organized dairy farms in Nagpur district, the findings may not fully represent AMR and virulence patterns in unorganized dairy farms or other geographical settings.

Nevertheless, these findings showed that the persistence of virulent and resistant *E. coli,* within dairy farm ecosystems, is influenced by several factors, with the observed seasonal and sample-type variations highlighting the interconnected roles of animals, humans, and the environment. Overall, the study demonstrates that livestock ecosystems can serve as important reservoirs of pathogenic *E. coli* and associated AMR determinants, underscoring the potential for cross-sectoral transmission at the animal, human, and environmental interface (86, 87). These findings reinforce the need for integrated One Health surveillance, strengthened farm hygiene and management practices, and judicious antimicrobial use to limit the persistence and dissemination of antimicrobial-resistant *E. coli* within livestock ecosystems (88).

## 5 Conclusion

Our study demonstrated a high occurrence of *E. coli* (70.06%) across animal, environmental, and human samples collected from organized dairy farms in Nagpur district, with the highest occurrence observed in environmental samples, highlighting the farm environment as an important reservoir for bacterial dissemination. The detection of *E. coli* across multiple interconnected compartments of the dairy-farm ecosystem reinforces the importance of a One Health perspective. Although *E. coli* O157 was not detected during the study period, the presence of Shiga toxin-associated genes, (*stx1* and *stx2*), particularly among animal-associated isolates, indicated the circulation of potentially pathogenic *E. coli* within the livestock environment. The observed seasonal variation in virulence profiles, especially during monsoon and winter, further highlighted the influence of temporal factors on their occurrence.

The antimicrobial susceptibility findings raised an additional public health concern, as resistance to commonly used antibiotics, the occurrence of MDR isolates, and the detection of multiple resistance determinants indicate the presence and potential circulation of resistant *E. coli* across different components of the livestock settings. Although the present study does not establish transmission pathways among these compartments, the observed seasonal variation, underscores the importance of incorporating temporal factors into farm-level AMR surveillance, and strengthening antimicrobial stewardship.

These findings support the need for coordinated surveillance strategies integrating animal, environmental, and human health perspectives to facilitate the early detection of emerging virulence and AMR patterns and guide targeted interventions at the farm level. Such approaches should be complemented by improved farm hygiene, effective manure and wastewater management, and safe water and feed practices to reduce opportunities for the persistence and dissemination of pathogenic and antimicrobial-resistant *E. coli*. In addition, systematic monitoring of antimicrobial use and evidence-based prescribing practices should be strengthened as key components of antimicrobial stewardship to minimize unnecessary antimicrobial exposure and selection pressure within livestock ecosystems.

Addressing the limitations of present study in future research will provide a basis for better understanding the epidemiology and transmission dynamics of virulent and antibiotic resistant *E. coli* within dairy-farm ecosystems. Larger and more diverse populations, longitudinal monitoring, quantitative assessment of antimicrobial use, and whole-genome sequencing would provide deeper insights into resistance and virulence determinants and to assess the genetic relatedness of the isolates.

Overall, this study highlights dairy farm ecosystems as important reservoirs and interfaces for the persistence and circulation of virulent and resistant *E. coli* across animal, human, and environmental compartments. These interconnected reservoirs underscore the need for coordinated One Health surveillance, improved farm and environmental management, and responsible antimicrobial use to limit the dissemination of resistant *E. coli* and strengthen AMR prevention and control.

## Data Availability

The original contributions presented in the study are included in the article/Supplementary material, further inquiries can be directed to the corresponding author.
