# Supplementary material for: Epidemiological insights into virulence and antimicrobial resistance of Escherichia coli across dairy farms in Nagpur district
Source: Front Vet Sci. 2026 Sep 23;13:1915714. doi: 10.3389/fvets.2026.1915714 (PMC13642211; doi:10.3389/fvets.2026.1915714)
Supplement: Supplementary file 1 [file Data_Sheet_1.pdf]

Supplementary Fig. S1. Colonies of *E. coli* on HiCrome E. coli agar

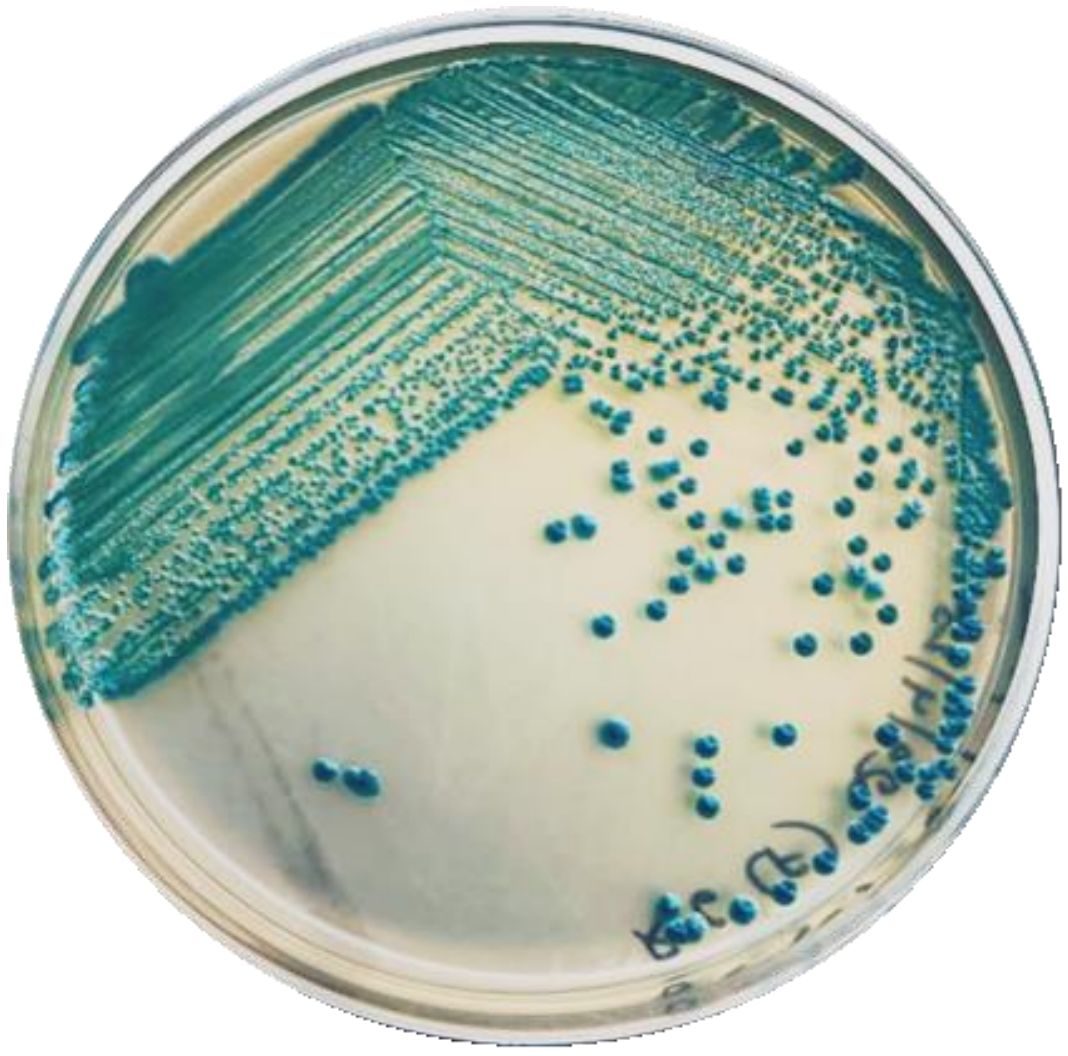

Supplementary Fig. S2 Colonies of *E. coli* O157 on EMB agar

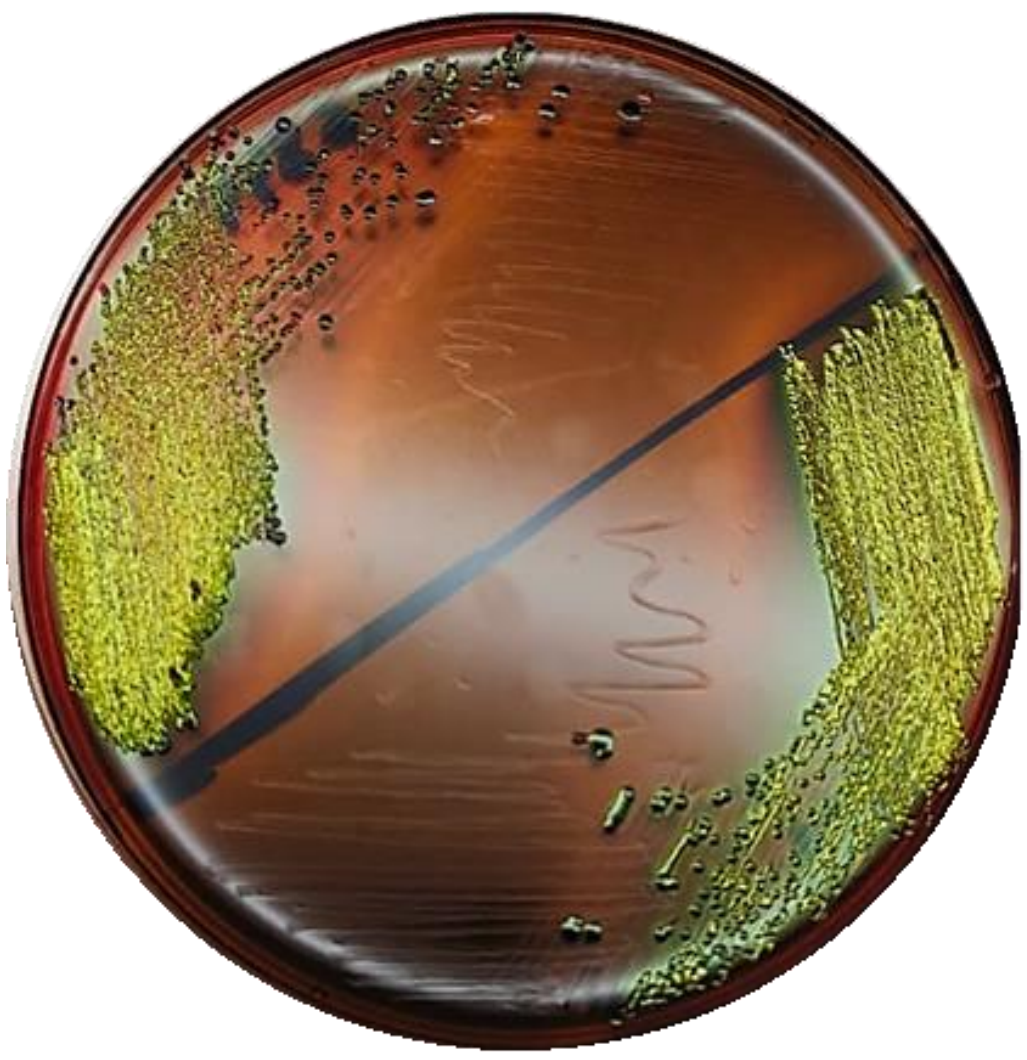

Supplementary Fig. S3 Gram's staining of *E. coli* isolates

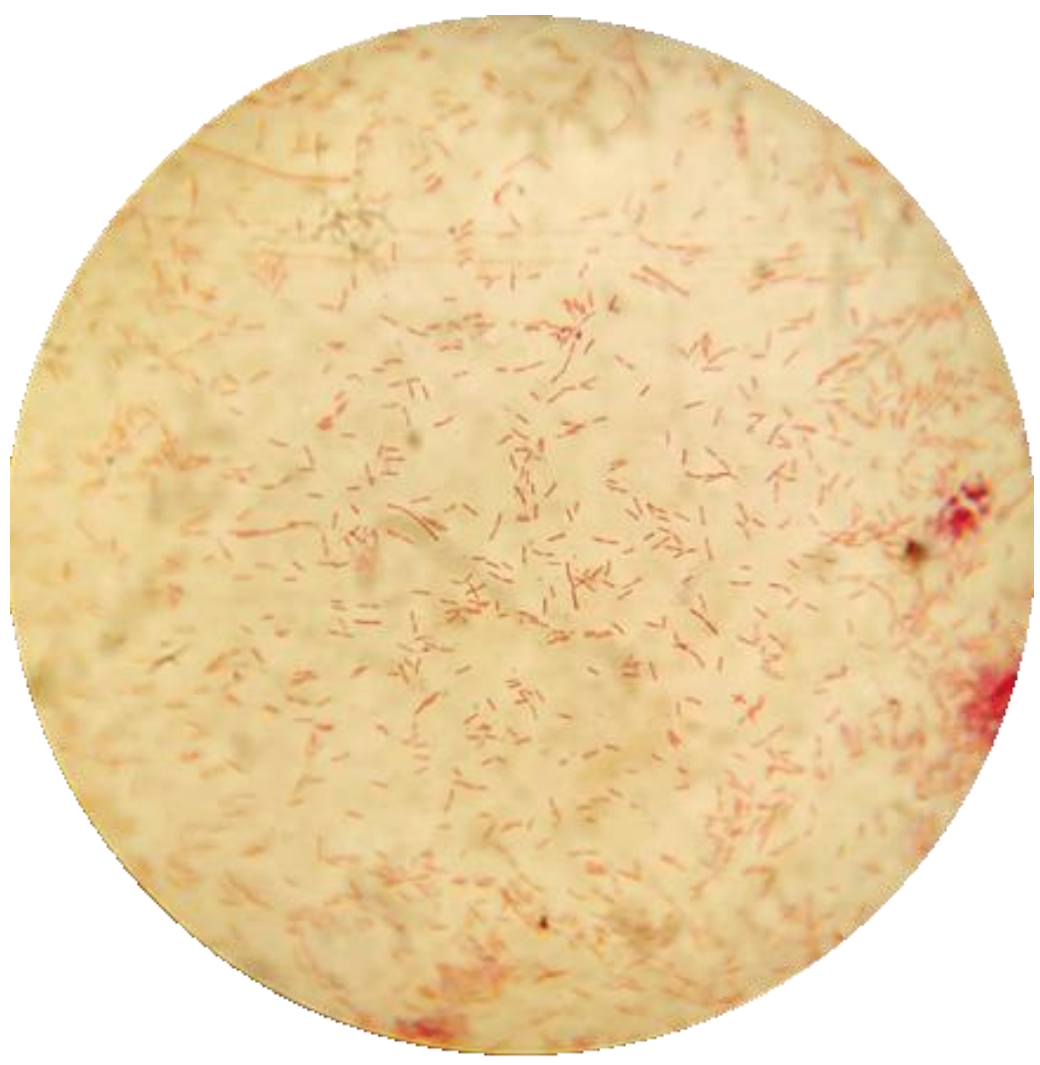

Supplementary Fig. S4 PCR profiling of the *uidA* gene of *E. coli*

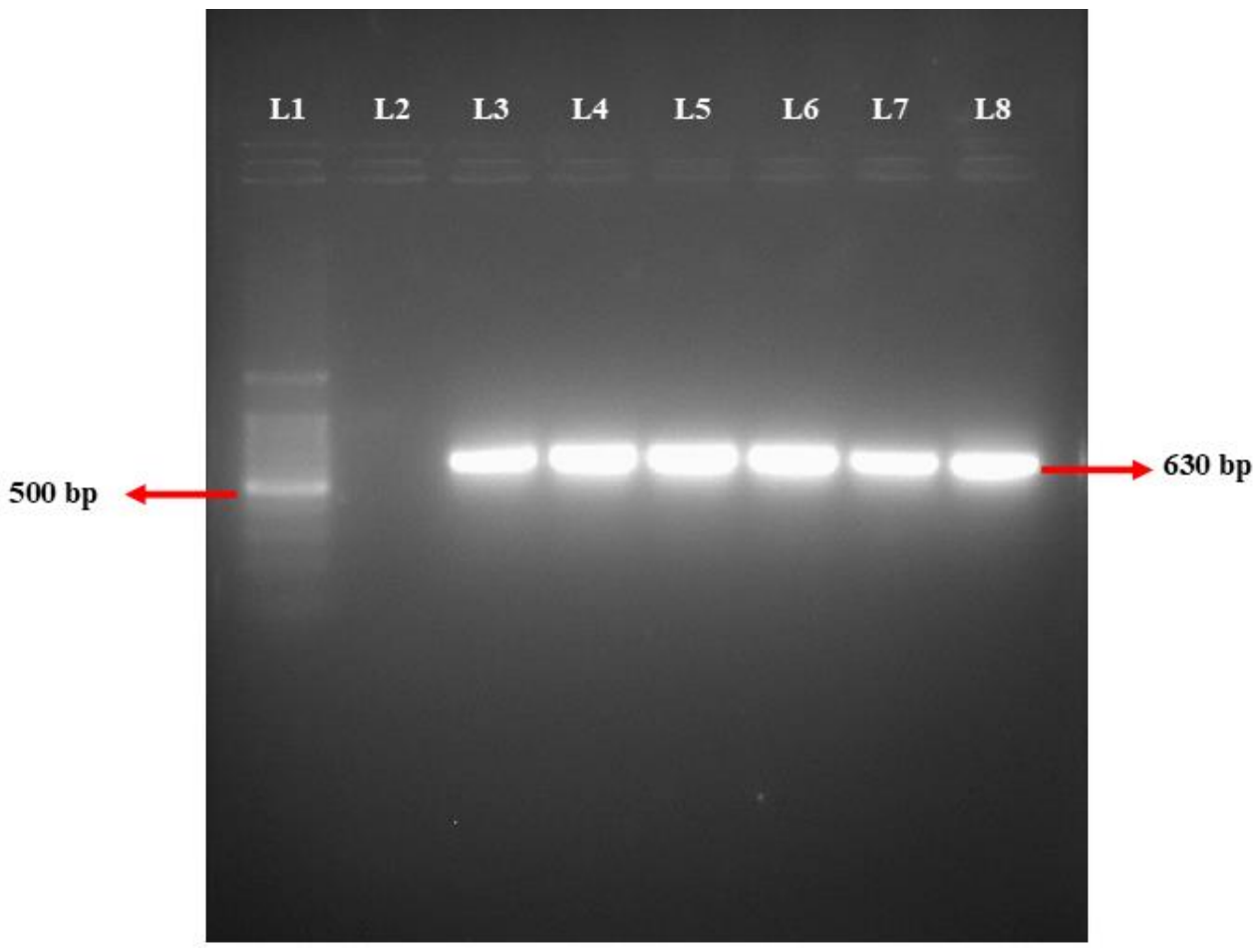

L1 – Ladder (100 bp)
L2 – Negative control
L3 – Positive control
L4 to L8 – Positive samples for the *uidA* gene

Supplementary Fig. S5 PCR detection of Shiga toxin virulence genes (*stx1* and *stx2*) in *E. coli*. **(A)** PCR amplification of the *stx1* gene, showing the expected amplicon size of **130 bp**. **(B)** PCR amplification of the *stx2* gene, showing the expected amplicon size of **346 bp**.

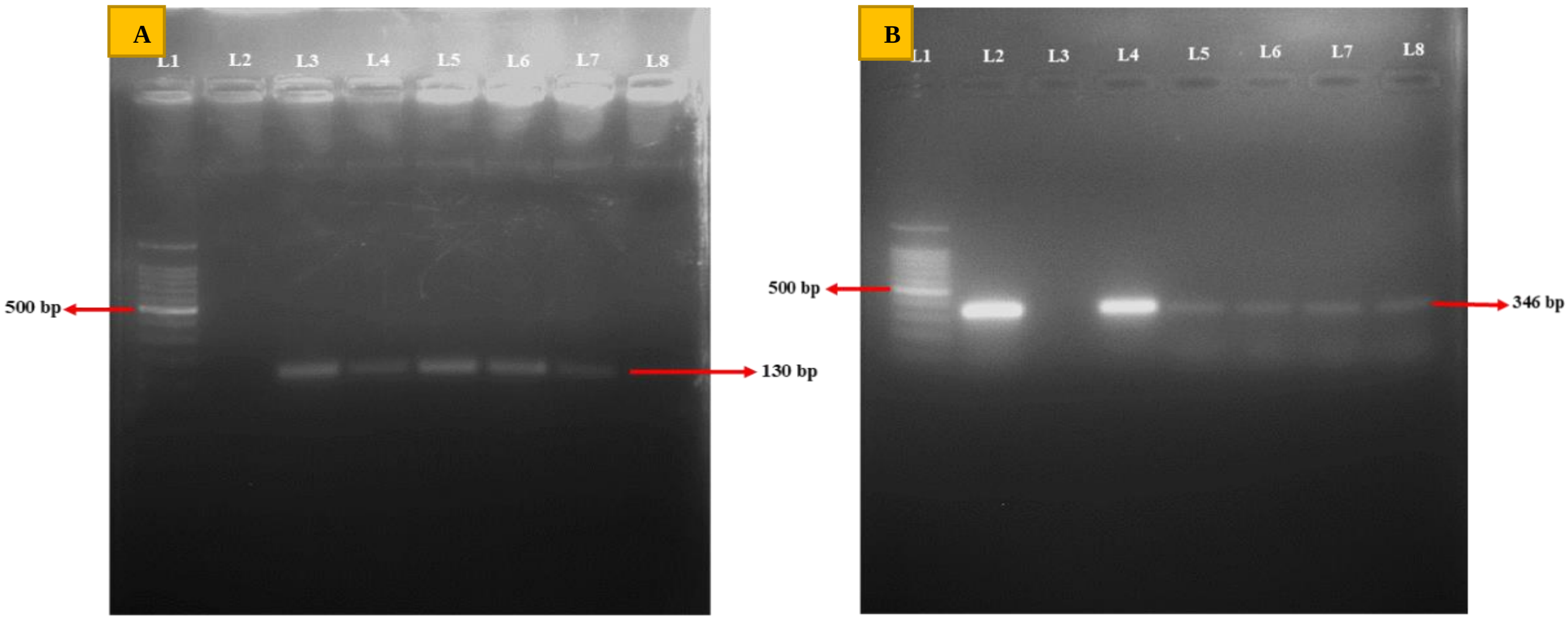

L1 – Ladder (100 bp)
L2 – Negative control
L3 – Positive control
L4 to L8 – Positive samples for the *stx1* gene

L1 – Ladder (100 bp)
L2 – Positive control
L3 – Negative control
L4 to L8 – Positive samples for the *stx2* gene

Supplementary Fig. S6 Antibiogram profile of *E. coli* isolates during the first collection (Summer)

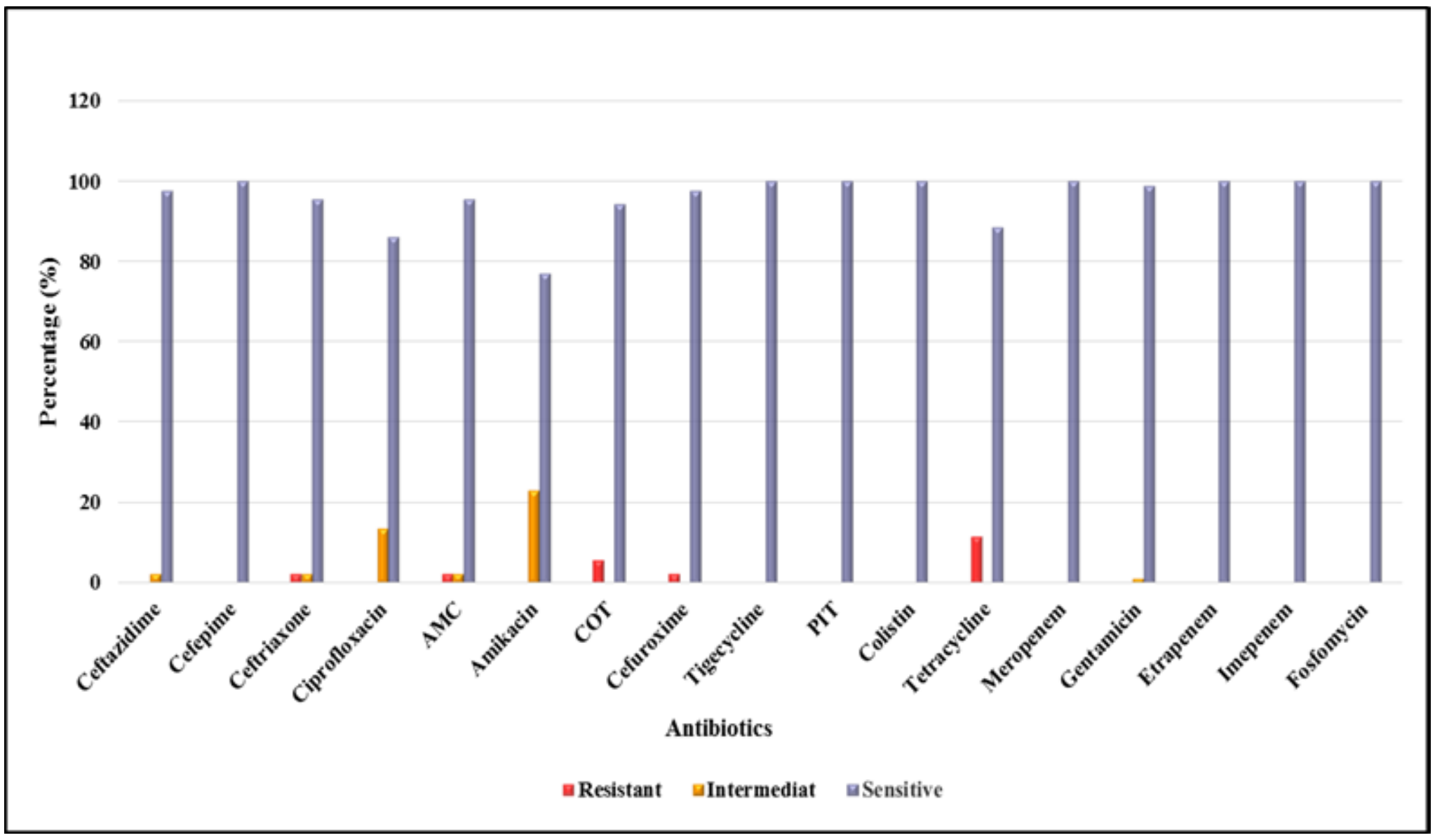

Supplementary Fig. S7 Antibiogram profile of *E. coli* isolates during the second collection (Early monsoon)

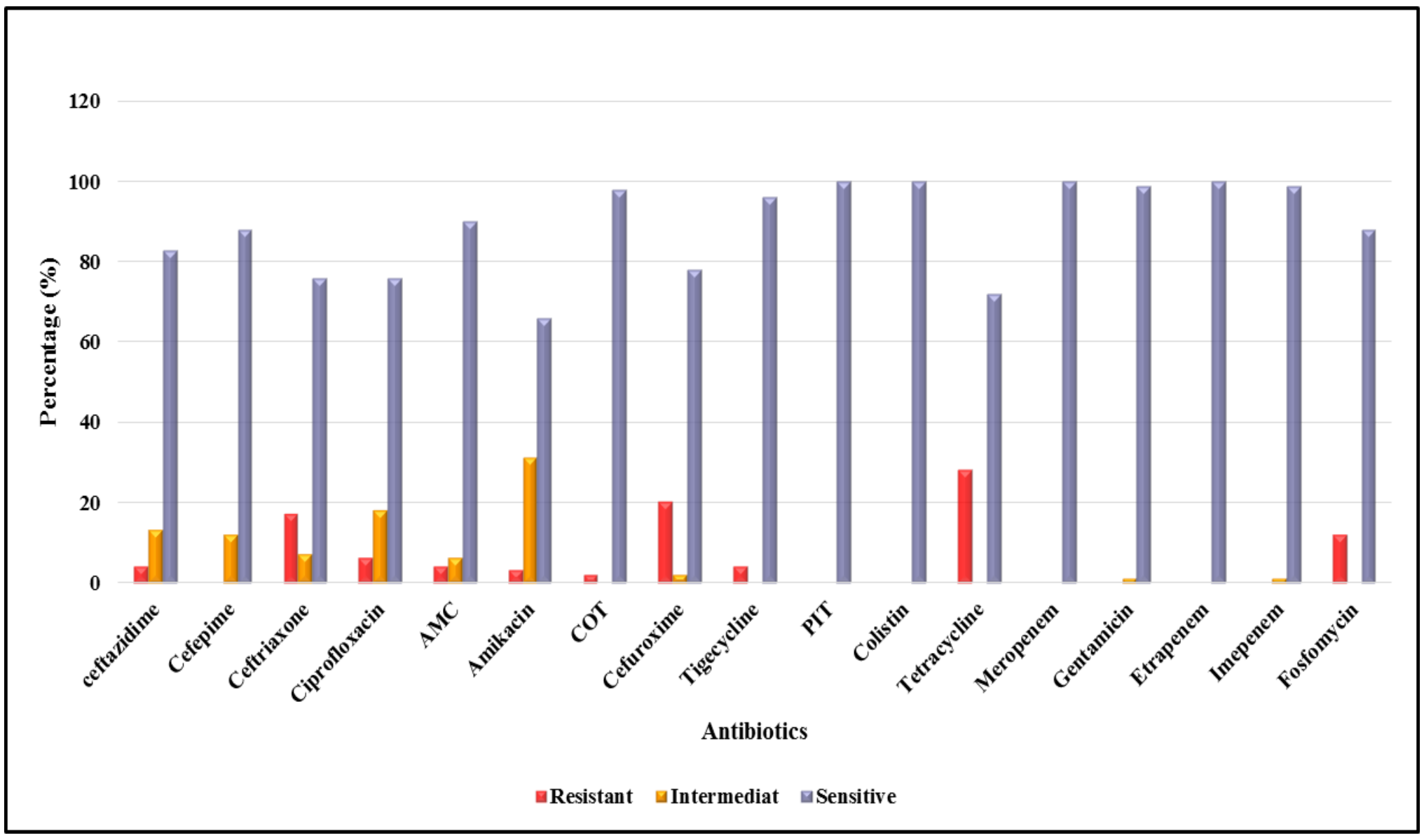

Supplementary Fig. S8 Antibiogram profile of *E. coli* isolates during the third collection (Peak monsoon)

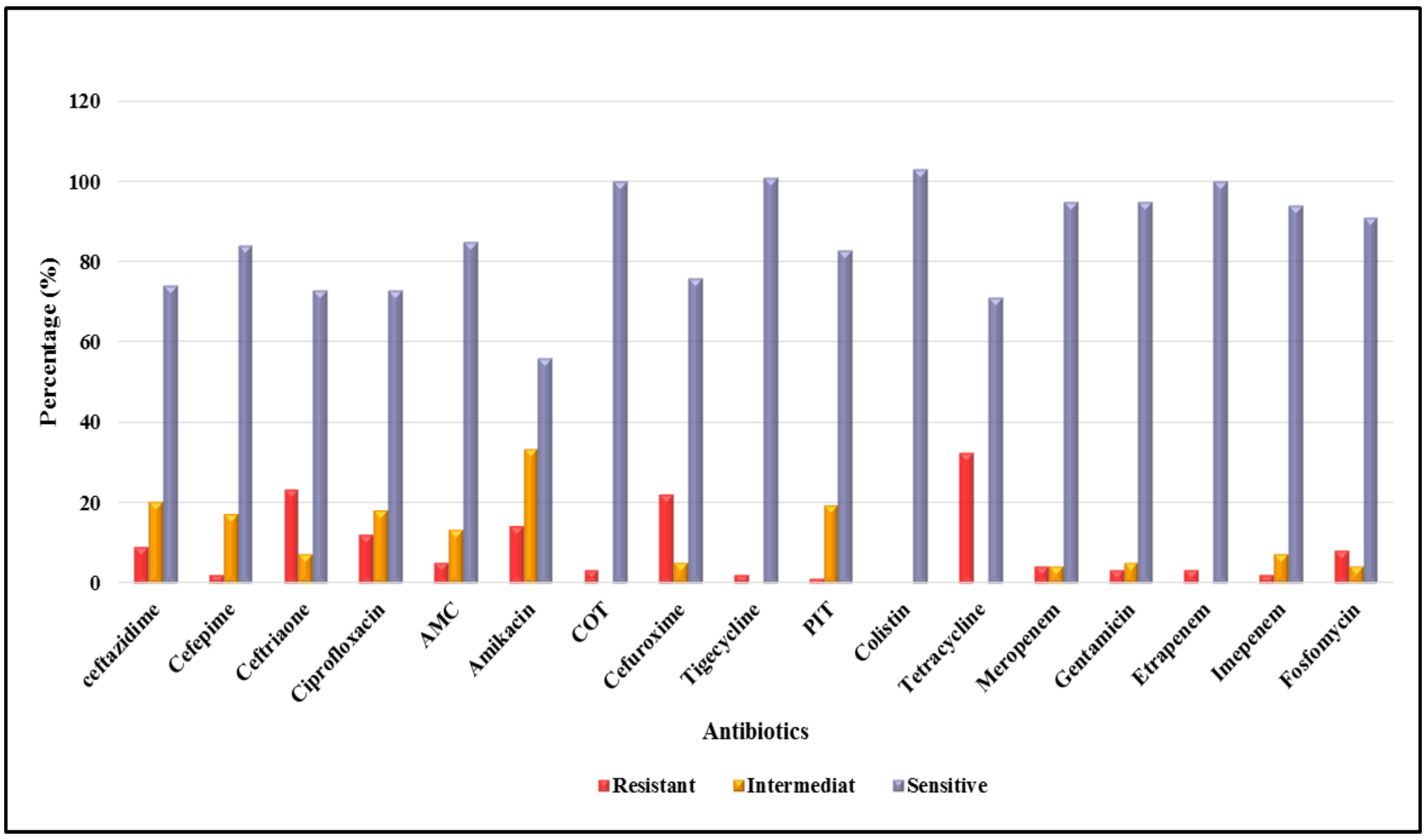

Supplementary Fig. S9 Antibiogram profile of *E. coli* isolates during the fourth collection (Winter)

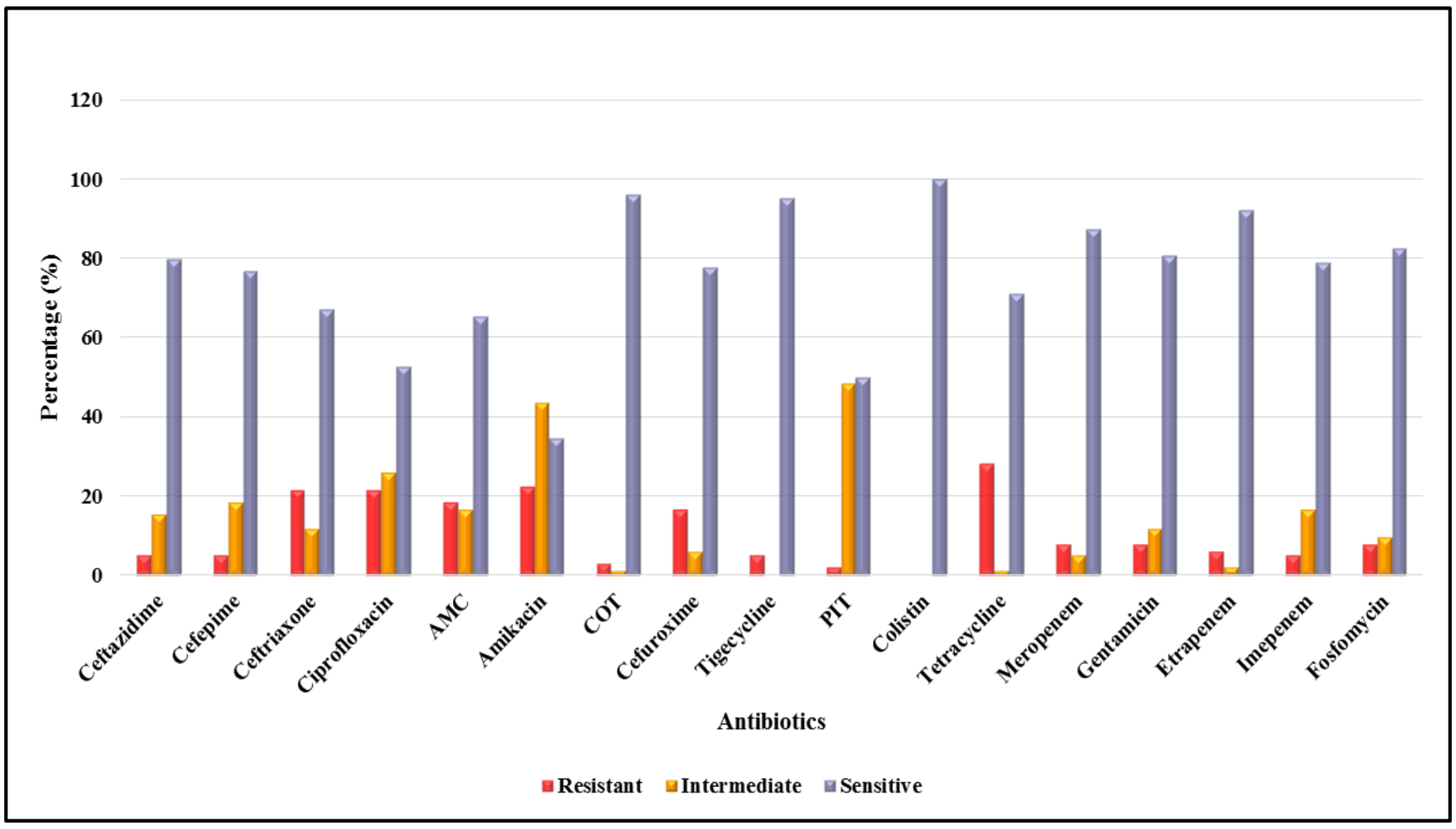

Supplementary Fig. S10 PCR detection of antimicrobial resistance (AMR) genes in *E. coli*.

(A) PCR amplification of the *tetA* gene, showing the expected amplicon size of **502 bp**. **(B)** PCR amplification of the *tetB* gene, showing the expected amplicon size of **930 bp**. **(C)** PCR amplification of the *blaTEM* gene, showing the expected amplicon size of **800 bp**. **(D)** PCR amplification of *blaCTX-M* gene variants, showing the expected amplicon sizes of **404 bp, 561 bp and 668 bp**. **(E)** PCR amplification of the *blaNDM* gene, showing the expected amplicon size of **404 bp**. **(F)** PCR amplification of the *AmpC* gene, showing the expected amplicon size of **634 bp**.

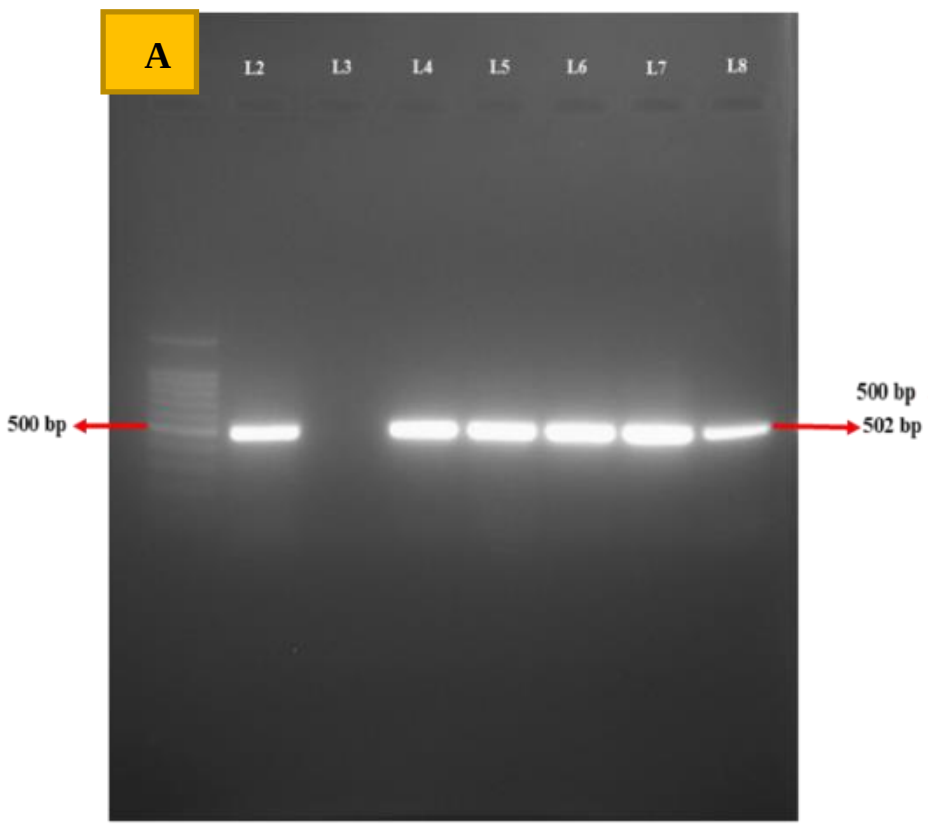

L1 – Ladder (100 bp)
L2 – Positive control
L3 – Negative control
L4 to L8 – Positive samples for the *tetA* gene

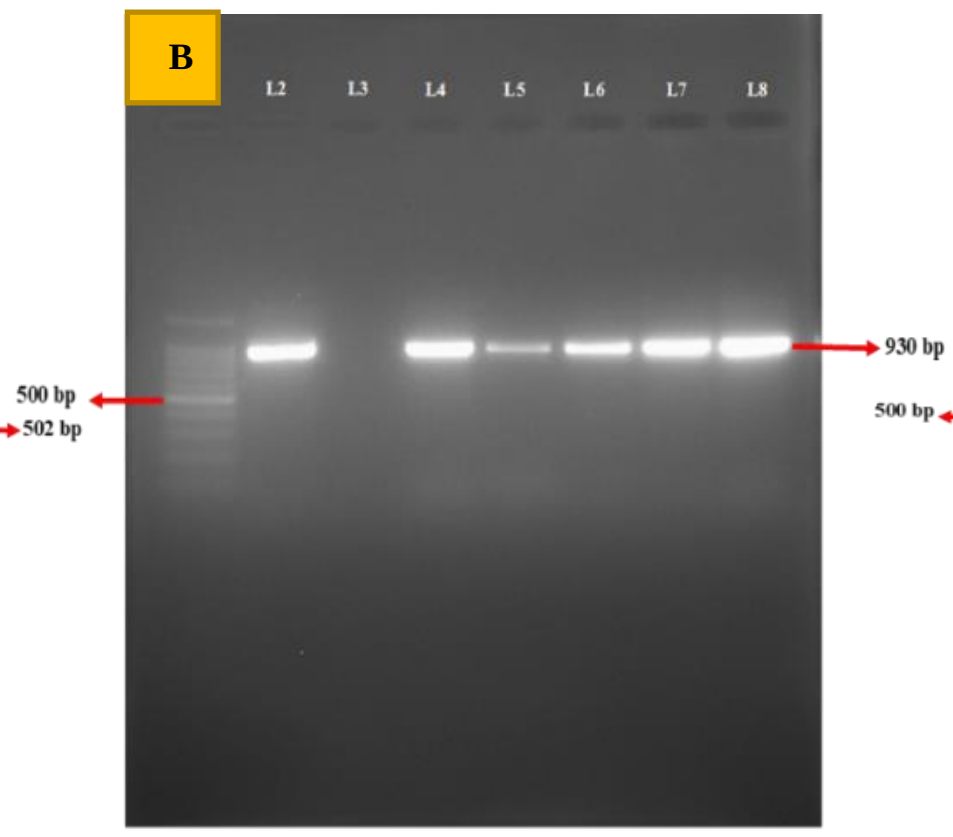

L1 – Ladder (100 bp)
L2 – Positive control
L3 – Negative control
L4 to L8 – Positive samples for the *tetB* gene

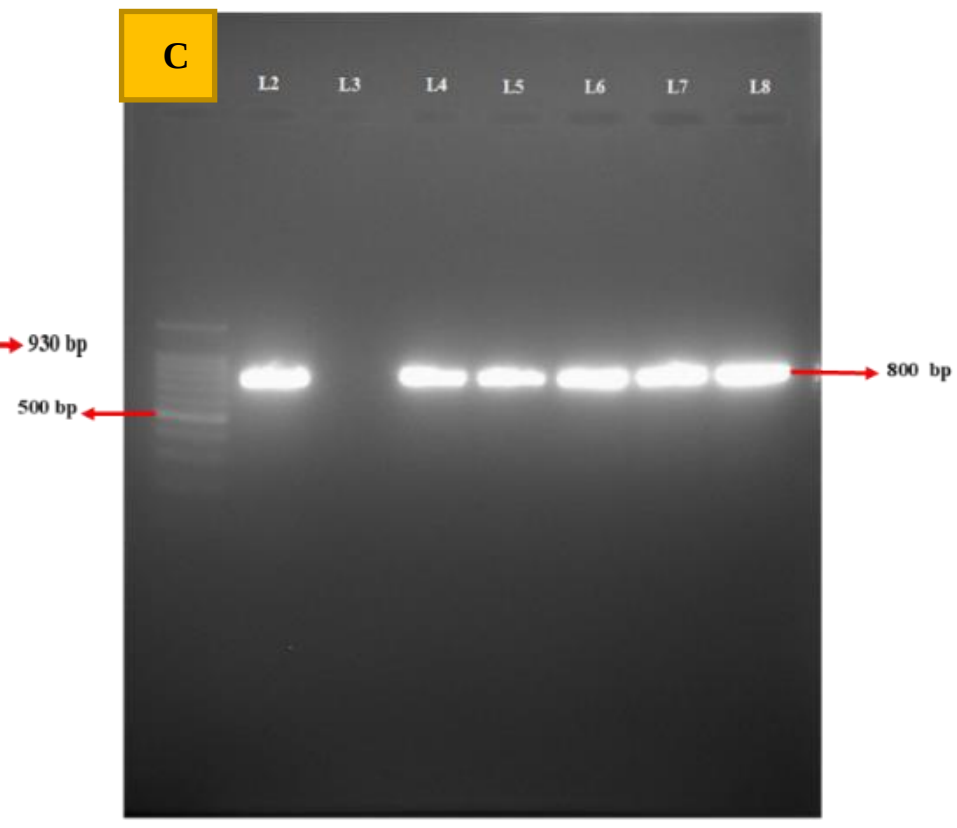

L1 – Ladder (100 bp)
L2 – Positive control
L3 – Negative control
L4 to L8 – Positive samples for the *blaTEM* gene

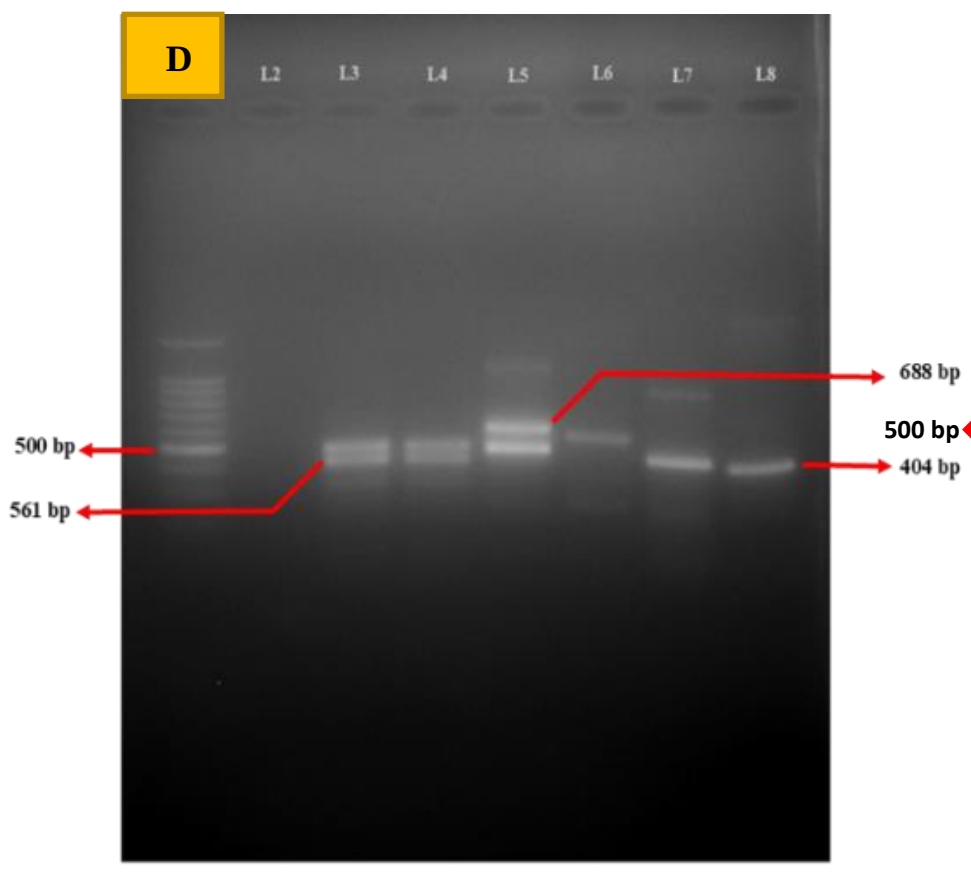

L1 – Ladder (100 bp)
L2 – Negative control
L3 to L8 – Positive samples for the *blaCTX-M* gene variants.

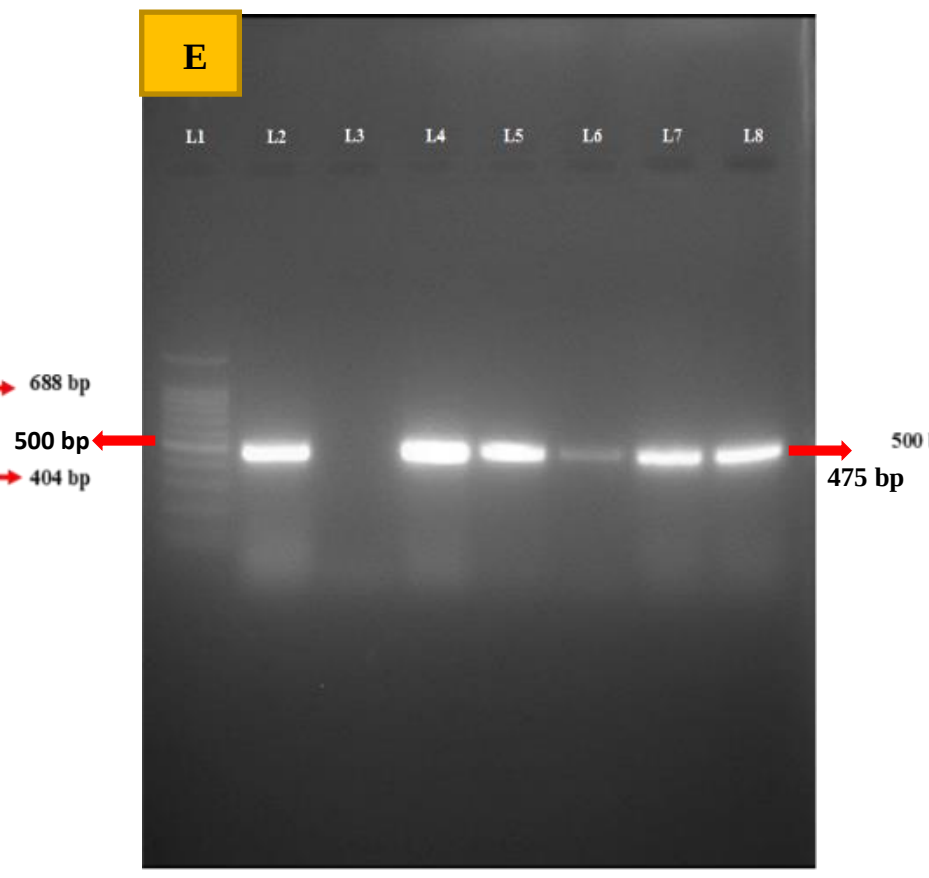

L1 – Ladder (100 bp)
L2 – Positive control
L3 – Negative control
L4 to L8 – Positive samples for the *blaNDM* gene

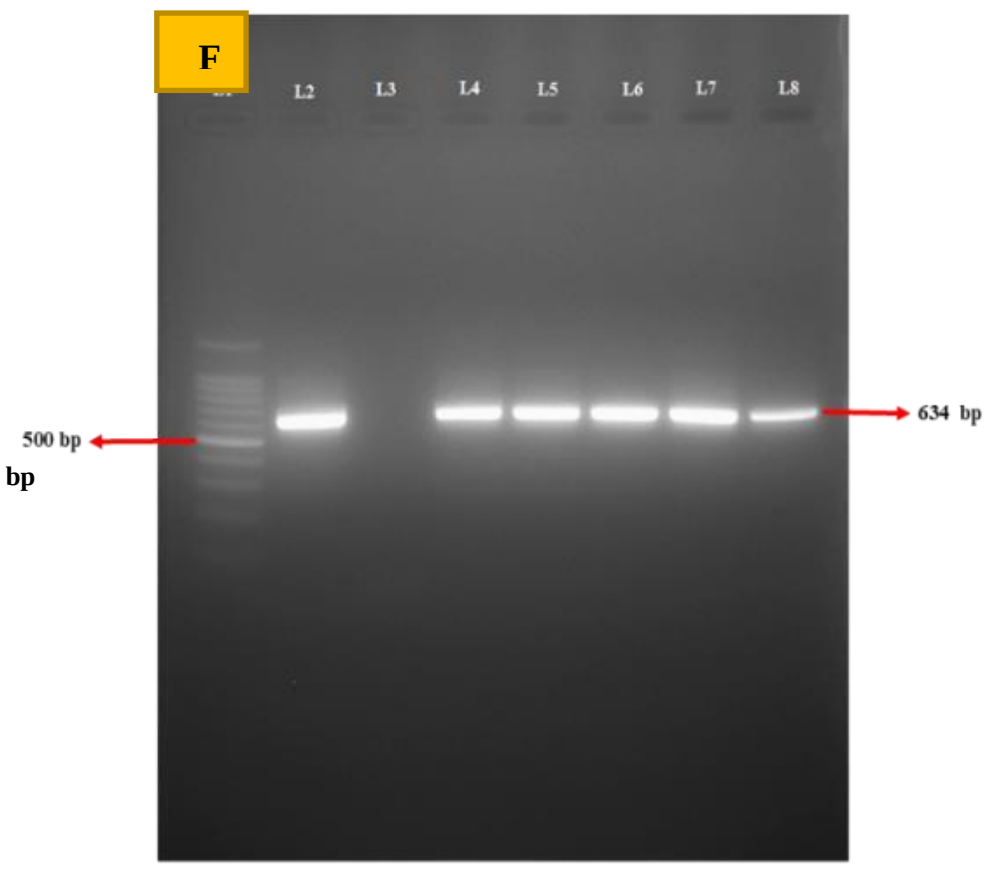

L1 – Ladder (100 bp)
L2 – Positive control
L3 – Negative control
L4 to L8 – Positive samples for the *AmpC* gene

Tables

Supplementary Table S1. Details of primers for identification and virulence genes of *E. coli*

Target gene	Primer	Sequence (5'-3')	Annealing temperature	Amplicon size	Reference
***uidA* (Species specific)**	**F**	CGCCGATGCAGATATTCG	52^0C / 40 sec (35 cycles)	630 bp	Modak *et al*., 2012
	**R**	GCTGTGACGCACAGTTCATAG			
***rfbE* (O157 antigen)**	**F**	CAGGTGAAGGTGGAATGGTTGTC	55^0C/30 sec (48 cycles)	296 bp	Bertrand and Roig, 2007
	**R**	TTAGAATTGAGACCATCCAATAAG			
stx1	**F**	GAAGAGTCCGTGGGATTACG	58^0C/30 sec (30 cycles)	130 bp	Favier *et al*., 2014
	**R**	AGCGATGCAGCTATTAATAA			
stx2	**F**	TTAACCACACCCCACCGGGCAGT	58^0C/30 sec (30 cycles)	346 bp	Favier *et al*., 2014
	**R**	GCTCTGGATGCATCTCTGGT			

Supplementary Table S2. Details of Primers for PCR targeting AMR genes *tetA, tetB, blaTEM, blaCTX-M, blaNDM* & *AmpC* variants

Target gene	Primer	Sequence (5'-3')	Annealing temperature	Amplicon size	Reference
**Multiplex I *TEM* Variants**	***TEM-1***	CATTTCCGTGTCGCCCTTATTC	55^0C/40 sec (35 cycles)	800 bp	Dallenne *et al*., 2010
	TEM-2	CGTTCATCCATAGTTGCCTGAC			
**Multiplex II *CTX-M* group1, group2 and group9**	***CTXM* Gp1 F**	TTAGGAARTGTGCCGCTGYA	55^0C/40 sec (35 cycles)	688 bp	Dallenne *et al*., 2010
	***CTXM* Gp 1-2 R**	CGATATCGTTGGTGGTRCCAT			
	***CTXM* Gp2 F**	CGTTAACGGCACGATGAC	55^0C/40 sec (35 cycles)	404 bp	
	***CTXM* Gp 1-2 R**	CGATATCGTTGGTGGTRCCAT			
	***CTXM* Gp9 F**	TCAAGCCTGCCGATCTGGT	55^0C/40 sec (35 cycles)	561 bp	
	***CTXM* Gp9 R**	TGATTCTCGCCGCTGAAG			
NDM-1	**F**	GGGCAGTCGCTTCCAACGGT	60^0C/30 sec (30 cycles)	475 bp	Ghatak *et al*., 2013
	**R**	GTAGTGCTCAGTGTCGGCAT			
AmpC	**F**	CCC CGC TTA TAG AGC AAC AA	52.3^0C/40 sec (35 cycles)	634 bp	Jacoby, G. A, 2009.
	**R**	TCA ATG GTC GAC TTC ACA CC			
tetA	**F**	GGCGGTCTTCTTCATCATGC	56.4^0C/1 min (35 cycles)	502 bp	Boerlin *et al*., 2005
	**R**	CGGCAGGCAGAGCAAGTAGA			
tetB	**F**	CATTAATAGGCGCATCGCTG	56.4^0C/1 min (35 cycles)	930 bp	Boerlin *et al*., 2005
	**R**	TGAAGGTCATCGATAGCAGG			

Supplementary Table S3 Antibiogram profile of *E. coli* isolates

ANTIBIOTIC	SENSITIVE (%)	INTERMEDIATE (%)	RESISTANT (%)
**Ceftazidime (CAZ 30)**	325 (82.48%)	51 (12.9%)	18 (4.56%)
**Cefepime (CPM 30)**	339 (86.04%)	48 (12.18%)	7 (1.77%)
**Ceftriaxone (CTR 30)**	302 (76.6%)	28 (7.1%)	64 (16.24%)
**Ciprofloxacin (CIP 5)**	279 (70.81%)	75 (19.03%)	40 (10.1%)
**Amoxycillin-clavulanic acid (AMC 30)**	326 (82.74%)	38 (9.64%)	30 (7.61%)
**Amikacin (AK 30)**	225 (57.1%)	129 (32.7%)	40 (10.15%)
**Co – Trimoxazole (COT 25)**	380 (96.4%)	1 (0.25%)	13 (3.29%)
**Cefuroxime (CXM 30)**	320 (81.21%)	13 (3.29%)	61 (15.4%)
**Tigecycline (TGC 15)**	383 (97.2%)	0 (0.0%)	11 (2.79%)
**Piperacillin / Tazobactam (PIT 100/10)**	322 (81.7%)	69 (17.51%)	3 (0.76%)
**Colistin sulphate (CS 10)**	394 (100%)	0 (0.0%)	0 (0.0%)
**Tetracycline (TE 30)**	294 (74.6%)	1 (0.25%)	99 (25.1%)
**Meropenem (MRP 10)**	373 (94.6%)	9 (2.28%)	12 (3.04%)
**Gentamicin (GEN 10)**	364 (92.3%)	19 (4.82%)	11 (2.79%)
**Ertapenem (ETP 10)**	383 (97.2%)	2 (5.07%)	9 (2.2%)
**Imipenem (IMP 10)**	362 (91.8%)	25 (6.34%)	7 (1.77%)
**Fosfomycin (FO 200)**	352 (89.3%)	14 (3.55%)	28 (7.1%)
